# Solvation-Guided Design of Fluorescent Probes for Discrimination of Amyloids

**DOI:** 10.1038/s41598-018-25131-2

**Published:** 2018-05-03

**Authors:** Kevin J. Cao, Kristyna M. Elbel, Jessica L. Cifelli, Jordi Cirera, Christina J. Sigurdson, Francesco Paesani, Emmanuel A. Theodorakis, Jerry Yang

**Affiliations:** 1 Department of Chemistry and Biochemistry, UC San Diego, La Jolla, CA 92093-0358 USA; 2 Departments of Pathology and Medicine, UC San Diego, La Jolla, CA 92093-0612 USA; 3 Departament de Química Inorgànica i Orgànica (Secció d’Inorgànica) and Institut de Química Teòrica i Computacional (IQTCUB), Universitat de Barcelona,c/Martí i Franquès 1-11, 08028 Barcelona, Spain

## Abstract

The deposition of insoluble protein aggregates in the brain is a hallmark of many neurodegenerative diseases. While their exact role in neurodegeneration remains unclear, the presence of these amyloid deposits often precedes clinical symptoms. As a result, recent progress in imaging methods that utilize amyloid-specific small molecule probes have become a promising avenue for antemortem disease diagnosis. Here, we present a series of amino-aryl cyanoacrylate (AACA) fluorophores that show a turn-on fluorescence signal upon binding to amyloids in solution and in tissue. Using a theoretical model for environmental sensitivity of fluorescence together with *ab initio* computational modeling of the effects of polar environment on electron density distribution and conformational dynamics, we designed, synthesized, and evaluated a set of fluorophores that (1) bind to aggregated forms of Alzheimer’s-related β-amyloid peptides with low micromolar to high nanomolar affinities and (2) have the capability to fluorescently discriminate different amyloids based on differences in amino acid composition within the binding pocket through exploitation of their solvatochromic properties. These studies showcase the rational design of a family of amyloid-binding imaging agents that could be integrated with new optical approaches for the clinical diagnosis of amyloidoses, where accurate identification of the specific neurodegenerative disease could aid in the selection of a proper course for treatment.

## Introduction

Progressive neurodegenerative diseases are one of the leading causes of death in the US^1^. While no cure currently exists for these diseases, progress has been made toward the treatment of late-stage symptoms such as memory loss and restlessness^2–8^. Substantial clinical evidence suggests that early diagnosis and intervention may provide the best chance for improving quality of life^9–11^. Unfortunately, the differential diagnosis between the various neurodegenerative disorders remains a difficult challenge, particularly during the early stages. For instance, clinical diagnosis of Alzheimer’s disease (AD) can only be tentatively made in late stages by combining neuropsychological evaluations and scanning technologies such as positron emission tomography, while definitive diagnosis is only confirmed from post-mortem examination of the brain^1,12^. Diagnostic criteria for other neurodegenerative diseases such as prion-related Creutzfeldt Jakob disease (CJD) are even less well-defined^12–16^. Thus, methods that can help diagnose and differentiate patients with neurodegenerative disorders, ideally pre-symptomatically, would be of great clinical value.

One of the defining characteristics of many neurodegenerative diseases is the presence of extracellular or intracellular deposits of misfolded protein aggregates known as amyloids^17,18^. While it is uncertain whether these amyloid deposits are causative of neurodegeneration or are a byproduct of the disease, it is widely accepted that their appearance in the brain precedes clinical symptoms by years, if not decades^19–21^. Although the general cross β-sheet organization of amyloids causes their macroscopic structural features to be very similar to one another, Nilsson, Hammarström, and co-workers have reported that conformation-sensitive fluorescent oligothiophenes can distinguish subtle differences in conformation of different strains of prions deposits *in vitro*^22,23^. However, at a molecular level, amyloids associated with different diseases are generally formed from different amyloidogenic proteins with distinct differences in their primary amino acid sequences^24,25^. Such molecular differences in composition could potentially be exploited for the differential diagnosis of various neurodegenerative diseases at pre-clinical stages where behavioral and neuropsychological criteria may be unreliable^26,27^.

We previously reported the design of amyloid-binding probes based on a fluorescent molecular rotor motif^28–30^. These probes are comprised of an electron donor conjugated to an electron acceptor, leading to an enhancement of the fluorescence emission upon binding to an amyloid^31,32^. For instance, we showed that an aminonaphthyl 2-cyanoacrylate (ANCA)-based probe (compound **2**) was capable of fluorescently labeling amyloid plaques associated with AD or prion disease in neuronal tissue with excellent specificity and reproducibility^33^. Intriguingly, upon binding to these amyloids, probe **2** exhibited a different color of fluorescence emission when bound to amyloids of different protein compositions^33,34^.

Inspired by these initial findings, here we measured the permittivity value of several amyloids using probe **2** as a spectroscopic ruler. We found that, indeed, probe **2** can differentiate between many of these amyloids, with the measured permittivity values indicating that they all generally contain molecular binding pockets that are relatively non-polar. In an effort to increase the environmental sensitivity of such fluorescent probes across such narrow range of permittivities, we synthesized and evaluated a set of amino-aryl cyanoacrylates (AACAs) with modifications in the length and polarizability of the fluorophores. *Ab initio* computational studies of probe solvation provided a theoretical molecular interpretation for the effects of different structural features on the difference in dipole moments between the excited and ground states of the probes, as well as provided estimates for the Onsager radius and torsional strain for each fluorophore^35^. Finally, we evaluated the capability of these new AACA probes to discriminate between β-amyloid (Aβ) and prion plaques in tissue and demonstrate proof-of-concept for using multiple probes to discriminate between amyloids with similar binding pocket polarities in a fluorescence fingerprint assay. These results, together with recent reports on the presence of amyloid deposits in optically accessible tissue such as in the eyes of human patients, suggest an exciting opportunity to develop non-invasive optical tools that allow detection and identification of different amyloidogenic proteins and their respective neurodegenerative diseases^36–44^.

## Results and Discussion

### Estimation of Binding Pocket Polarity of Different Amyloid Aggregates

**Figure 1:**
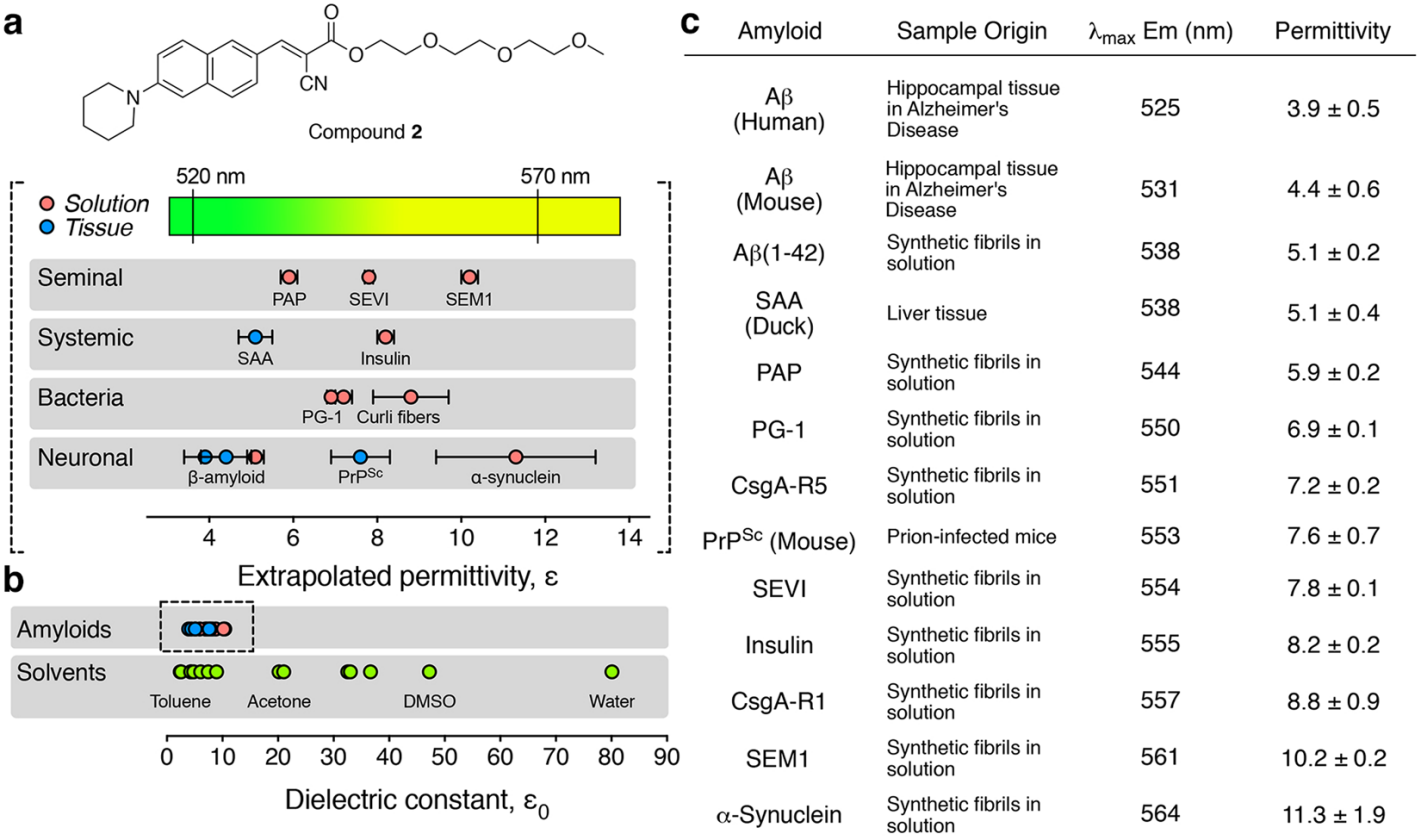
Fluorescence discrimination of amyloids using ANCA compound **2** as a spectroscopic ruler. (**a**) Structure of compound **2** and the maximal fluorescence emission wavelength observed (and associated extrapolated environment polarities) when this compound is bound to different amyloids. (**b**) Comparison of extrapolated permittivities of amyloid binding pockets relative to common solvent permittivities. (**c**) Tabulated emission and extrapolated permittivity values for compound **2** bound to different amyloid aggregates. See supporting information for description of amyloid species analysed.

Molecular dynamics simulations have indicated that amyloid-binding probes such as thioflavin T (ThT) and probe **2** adopt different conformations upon binding to amyloids due to the influence of different amino acid residues in the β-sheet region^45,46^. We theorized that differences in amino acid sequences of amyloidogenic proteins could lead to variations in the polarity of the microenvironment within the binding pocket and, thus, affect the fluorescence properties of environmentally sensitive probes such as **2**. With this in mind, we used probe **2** as a spectroscopic ruler to estimate the range of polar environments found in the binding pockets of 11 different proteins/peptides in amyloid form. This set of amyloid samples included natural tissue deposits as well as synthetic or recombinant peptide and protein aggregates in solution (Fig. 1). To evaluate whether there were significant differences in binding pocket polarities between amyloids of different biological origin, we selected representative examples of amyloids associated with neurodegenerative (e.g., Aβ, PrP^Sc^, α-synuclein) and systemic diseases (e.g., SAA, insulin), in addition to natural bacterial (e.g., PG-1, CsgA-R1, CsgA-R5) and seminal amyloids (PAP, SEVI, SEM1).

The fluorescence emission (λ_max_) of probe **2** when bound to 11 different amyloid species in tissue or in solution is provided in Fig. 1. We observed that the dynamic range of colors (Fig. 1a) and corresponding emission wavelength (λ_max_, Fig. 1c) of compound **2** bound to these amyloids spanned ~40 nm. In order to relate the observed color of fluorescence emission from probe **2** to an intrinsic property of the amyloid binding pocket, we approximated that its environmental sensitivity can be described by the theoretical model introduced by Lippert and Mataga^33,47–49^. This model provides a quantitative approximation of the relationship between the observed Stokes Shift (in wavenumbers), ∆$\tilde{\nu }$, of a fluorogenic species with the local polar environment, described by the relative permittivity (i.e., dielectric constant), *ε*, and the refractive index, *n*, as shown in equation (1):

1 $${\rm{\Delta }}\tilde{\nu }=\frac{2{({\mu }_{e}-{\mu }_{g})}^{2}}{hc{a}^{3}}[(\frac{\varepsilon -1}{2\varepsilon +1})-(\frac{{n}^{2}-1}{2{n}^{2}+1})]+C$$

Here, the difference in excited versus ground state dipole moments, (*μ*_e_ − *μ*_g_), and the Onsager radius, *a*, are properties inherent to the fluorophore, *h* and *c* are Planck’s constant and the speed of light, respectively, and *C* is the observed Stokes shift of the fluorophore in a non-solvatochromic environment^47–49^.

Since we previously found that the absorption properties of molecule **2** was not significantly affected by the environmental permittivity and that the effects of environmental permittivity dominated any contribution of environmental refractive index on fluorescence emission^33^, the fluorescence emission properties of probe **2** could then be described by equation (2), where ${\tilde{\nu }}_{{\rm{em}}}$ is the emission in wavenumbers and *C*_1_ is the observed emission in the absence of environment-dependent processes that affect fluorescence emission (see Supplementary Information for additional details on the derivation of equation (2)):

2 $${\tilde{\nu }}_{{\rm{em}}}=\frac{1}{{\lambda }_{{\rm{em}}}}=\frac{2{({\mu }_{e}-{\mu }_{g})}^{2}}{hc{a}^{3}}(\frac{\varepsilon -1}{2\varepsilon +1})+{C}_{1}$$

Equation (2), thus, made it possible to generate a calibration curve that relates the observed wavelength of fluorescence emission (λ_em_) of probe **2** and the single variable of environmental permittivity, *ε*, by measurement of fluorescence in different solvents with known permittivity (see Supplementary Figure 1). This calibration curve then allowed us to estimate the relative permittivities of the binding pocket for compound **2** on different amyloids from the observed fluorescence emission (λ_max_) values of bound compound **2** (Fig. 1c). These studies reveal that the binding pockets for probe **2** on amyloids spans a remarkably narrow range of relative permittivities and are generally quite non-polar (Fig. 1b). We, therefore, sought to design fluorescent amyloid-discriminating probes that exhibit enhanced environmental sensitivity, as compared to **2**, in relatively non-polar environments.

### Design of New Amino-Aryl CyanoAcrylate (AACA) Probes

Central to the fluorescent molecular rotor motif is the electronic coupling of an electron donor with an electron acceptor group through a π-conjugated network. In the specific structure of probe **2**, the amine donor is connected to a cyanoacrylate acceptor via the π network of a naphthalene unit. In this configuration, the probe responds to photoexcitation with an intramolecular charge transfer between the donor and acceptor to generate a charged excited state (S_1_) dipole. Relaxation from this state can occur via a combination of fluorescence emission and non-fluorescent mechanical de-excitation (e.g., rotation across σ-bonds). The latter pathway decreases upon restricted rotation due to amyloid binding leading to a significant increase of the emission quantum yield. On the other hand, the emission wavelength depends on the energy difference between the ground and excited state dipoles that, in turn, can be affected by the polarity of the microenvironment. Based on the Lippert-Mataga model, the inherent sensitivity of a fluorophore to polarity is governed by a molecular term (i.e., the first term in equations (1) and (2)) defined by the difference in ground and excited state dipoles, (*μ*_e_ − *μ*_g_), as well as the Onsager cavity radius of solvation, *a*, occupied by the fluorophore in the solvation field.

**Figure 2:**
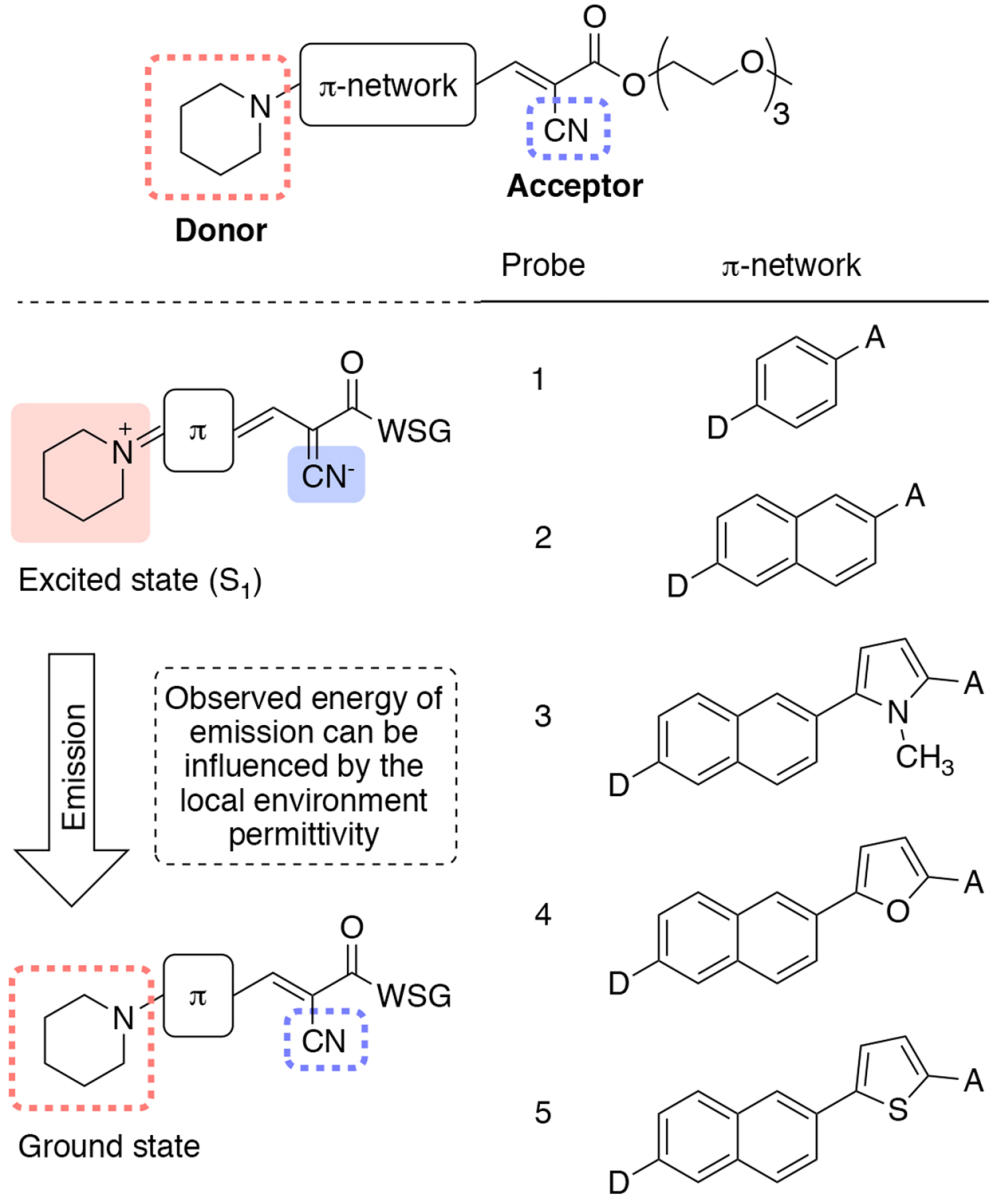
Structure of amino-aryl cyanoacrylate (AACA) fluorophores and the proposed mechanism of environment sensitivity. WSG = water solubilizing group.

Guided by this theoretical model, we sought to identify general criteria that would allow the rational design of fluorescent probes with enhanced environmental sensitivity (i.e., improved capability to differentiate the binding pocket microenvironments of different amyloids). To this end, we synthesized and examined the fluorescence properties of a set of five related fluorophores (Fig. 2, see Supplementary Information for details of the syntheses). In compounds **1–5** we varied the number of aryl groups between the electron donor and acceptor [i.e., probe **1** (1 ring), probe **2** (2 rings), and probes **3–5** (3 rings)], in order to examine the effects of the π-network length on environmental sensitivity within the range of local environments available in the binding pockets of amyloids. In compounds **3–5** we altered the heteroaromatic ring in order to evaluate the effect of electron donating heteroatoms on *μ*_e_, *μ*_g_ and *a* (equation (1)).

### Spectroscopic Properties of Fluorescent Probes 1–5

**Figure 3:**
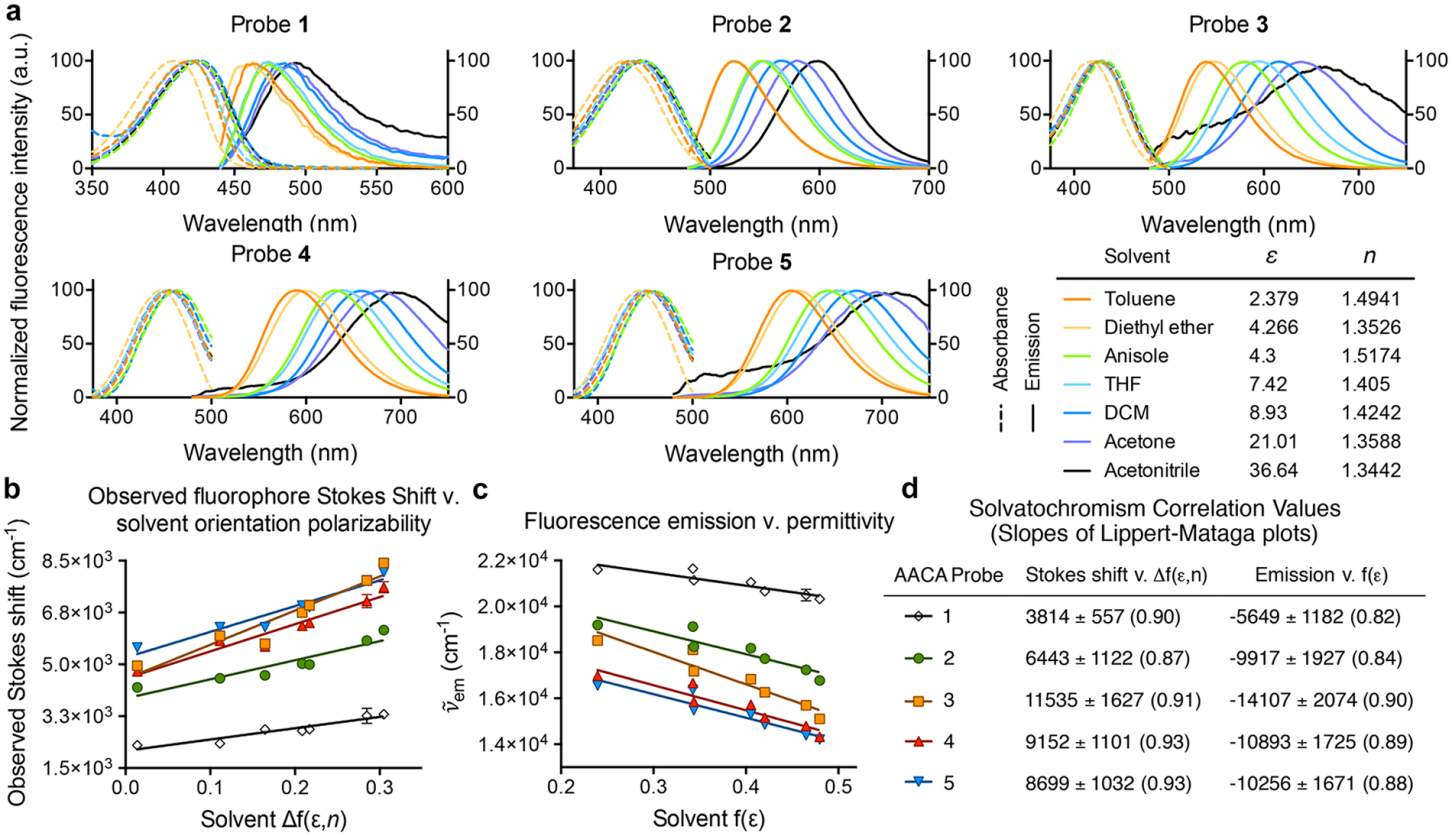
Solvatochromic properties of AACA probes **1–5** in polar aprotic solvents. (**a**) Absorption and fluorescence emission spectra of the fluorophores in select solvents show positive solvatochromism. (**b**) Lippert-Mataga plot of the observed stokes shift (in wavenumbers) as a function of the solvent orientation polarizability, Δf(*ε*, *n*). (**c**) Plot of solvatochromism fit to equation (2), where the observed fluorescence emission (in wavenumbers) as a function of solvent permittivity supports that the emission bands are not highly dependent on the environment refractive index. (**d**) Tabulated slopes of the Lippert-Mataga plots (**b**) and (**c**) and the linear regression coefficient (R^2^) in parenthesis. Slope values are in wavenumbers per unit permittivity.

The absorption and fluorescence emission spectra of AACA probes **1–5** were measured in an array of aprotic solvents (Fig. 3a, see also Supplementary Table 1 for tabulated fluorescence properties). Here, we excluded protic solvents from this study in order to avoid the potential influence of intermolecular forces such as hydrogen-bonding between protic solvent molecules and the probes that could mask the effect of solvent permittivities on the spectroscopic properties of the probes. We found that the absorbance bands were essentially independent (or weakly dependent) on the solvent permittivity. Additionally, the fluorescence emission wavelength exhibited a significant bathochromic shift with the increasing polarity of the solvent, defined as the solvent orientation polarizability Δf(*ε*, *n*)^50^:

3 $${\rm{\Delta }}f(\varepsilon ,n)=(\frac{\varepsilon -1}{2\varepsilon +1})-(\frac{{n}^{2}-1}{2{n}^{2}+1})$$

Furthermore, the observed linear relationship between the Stokes shifts of compounds **1–5** as a function of the solvent orientation polarizability (Fig. 3b) supports that equation (1) provides a reasonable theoretical model for the dependence of the fluorescence properties of these compounds on environmental polarity. Moreover, Fig. 3c demonstrates that the refractive index of the environment does not contribute significantly to the observed Stokes shift of compounds **1–5**, making it possible to relate the observed fluorescence wavelength of emission to the relative permittivity of the environment as given in equation (2). The slopes of the regression lines in Fig. 3B and C (tabulated in Fig. 3d) represent the relative environmental sensitivity for each molecule (see also Supplementary Figure 2 and Supplementary Table 2 for additional regression analyses).

We observed that probe **1**, harboring a benzene π-network, had the weakest environmental sensitivity of all probes tested (Fig. 3c). This observation can be explained by considering that shorter molecules produce smaller magnitudes of dipole moments in the S_1_ state, and, thus, smaller difference in (*μ*_e_ − *μ*_g_). On the other hand, the naphthalene-containing probe (compound **2**) was found to have a comparable sensitivity to the naphthalenyl-furan (probe **4**) and the naphthalenyl-thiophene (probe **5**) probe. We attributed this finding to the fact that while the difference in dipole moments between excited and ground states are expected to be larger for longer probes **4** and **5** compared to probe **2**, the increased length of **4** and **5** also increases their Onsager radii compared to compound **2**. This result highlights the effect of increasing length of the π-network in AACA probes on increasing both the excited and ground state dipole moments and radius of the fluorogenic core, which have opposing consequences on environmental sensitivity according to equations (1) and (2). Among all compounds tested, the naphthalenyl-pyrrole (probe **3**) had the greatest environmental sensitivity, suggesting that the effects of the π-network on the length of the dipole moment and the Onsager radius may not be the only structural features to consider for developing environmentally sensitive fluorescent probes. We, therefore, sought to gain additional molecular insight into the relationship between structure and environmental sensitivity of probes **1–5** using computational techniques.

### Structural and Computational Analyses Reveal New Insights on the Environmental Sensitivity of AACA Probes

**Figure 4:**
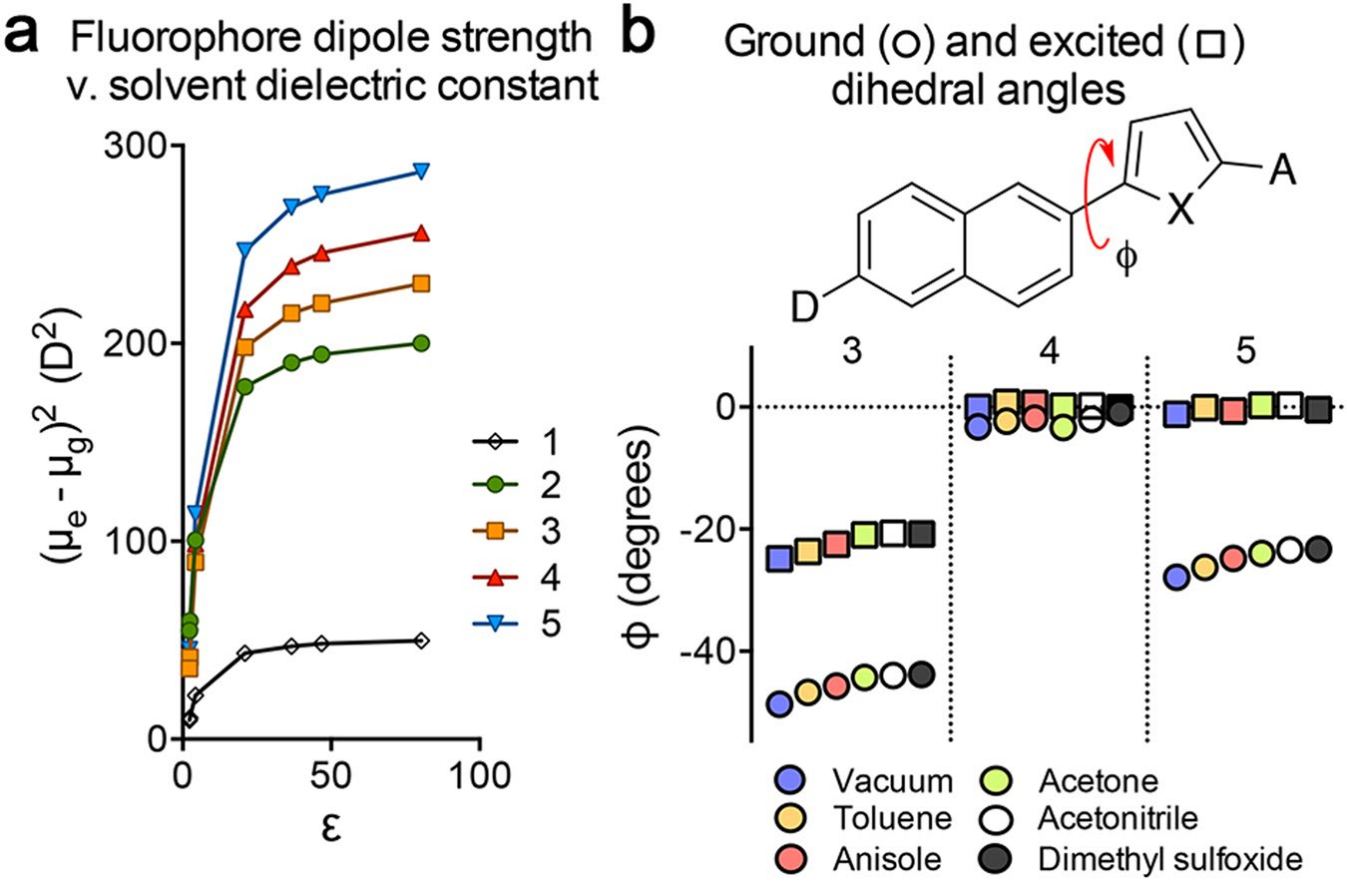
Calculated molecular dipole and torsional strain for AACA probes as a function of the environment polarity. (**a**) Square of the difference of the ground and excited states dipole moments as a function of the environment dielectric constant (*ε*). (**b**) Calculated dihedral angles between the naphthalene and heterocyclic aromatic rings of the ground (○) and excited (□) states for AACA probes **3–5** in polar aprotic solvents. X = NMe (AACA **3**), O (AACA **4**), and S (AACA **5**).

The Lippert-Mataga plots in Fig. 3b and c indicate that probe **3** contains the strongest environmental sensitivity (i.e., steepest slope of the regression lines). To gain further insight into this observation, we performed density function theory (DFT) calculations of probes **1–5** in vacuum and solvated in various aprotic solvents (Fig. 4). Since environmental sensitivity is typically inferred from experimental plots (e.g., Fig. 3b and c), we directly calculated the dipole moments and the Onsager radii of probes **1–5** in order to evaluate the predictive value of the Lippert-Mataga model (see Supplementary Table 3 for calculated dipole values). Plotting the *ab initio* calculated square of the difference in dipole moments between the excited and ground states [(*μ*_e_ − *μ*_g_)^2^] as a function of the solvent dielectric constant (Fig. 4a) indicated that the three heterocyclic aromatic probes (**3–5**) had the greatest change in dipole strength [(*μ*_e_ − *μ*_g_)^2^] compared to probes **1** and **2** as solvent polarity (as approximated by dielectric constant, *ε*) increased. In contrast to the experimental data for solvatochromism shown in Fig. 3a, these computational studies suggested that the naphthalenyl-thiophene conjugate (probe **5**) is expected to produce the greatest difference in excited/ground state dipole moments at large environment polarity. Thus, in theory, probe **5** would be expected to exhibit the greatest environmental sensitivity of all probes tested according to equation (1). However, Fig. 4a also shows that in relatively non-polar environments (such as within the binding pocket of an amyloid), the difference in (*μ*_e_ − *μ*_g_)^2^ rapidly decreases for all probes **1–5** as the surrounding dielectric environment approaches zero (e.g., a vacuum). As a result, in low dielectric environments, we would not expect large differences in environment sensitivity for probes **3–5** based on differences in excited/ground state dipole moments alone. Additional DFT calculations determined the radius of probe **3** to be 8.59 Å, compared to 9.01 Å for probe **4** and 9.23 Å for probe **5**. Considering equation (1), expansion of the size of the solvation cavity is expected to reduce the environment sensitivity. Thus, taken together, these results suggest that probe **3** would be expected to have the strongest sensitivity to polarity than probes **4** and **5**.

In order to provide additional insight into the larger than expected environmental sensitivity of probe **3**, we computationally evaluated the conformational dynamics of these donor-acceptor fluorophores, which are known to form twisted-intramolecular charge transfer (TICT) states^28,31^. The TICT state is known to be stabilized by highly polar environments^28,51^. We hypothesized that the enhanced environmental sensitivity of the naphthalenyl-pyrrole probe **3** could arise from torsional strain along the presumed freely rotating single bonds between the electron donor and acceptor of the AACA probes. DFT calculations provided an estimate for the average dihedral angle in the ground and excited states for compounds **3–5** between the naphthalene and the heterocyclic aromatic ring in various dielectric environments (Fig. 4b).

In the ground state, probes **3** and **5** were found to deviate significantly from planarity, with probe **3** being the most twisted with an average torsional angle of ϕ = 45.5° across all solvents. Upon photoexcitation, the excited state of probe **3** becomes more coplanar than the ground state, with an average torsional angle of ϕ = 22.2°. In contrast, probe **4** and probe **5** exhibited essentially fully planar photoexcited states, with average dihedral angles of ϕ = 0.265° and ϕ = 0.298°, respectively (see Supplementary Table 4 for additional values from torsional angle calculations). These results suggest that the emissive properties of the probe **3** are not solely dependent on the polarity of the environment alone. As seen with thioflavin T, conformational strain between the donor and acceptor may be compensated by additional interactions between the fluorophore and environmental molecules (such as solvent or specific amino acid side chain residues within the binding pocket of amyloids), which can further stabilize the excited state and enhance environmental sensitivity^46^.

### AACA Probe 3 Exhibits Superior Fluorescence Amyloid Discrimination Capability in *Ex Vivo* Neuronal Tissue

**Figure 5:**
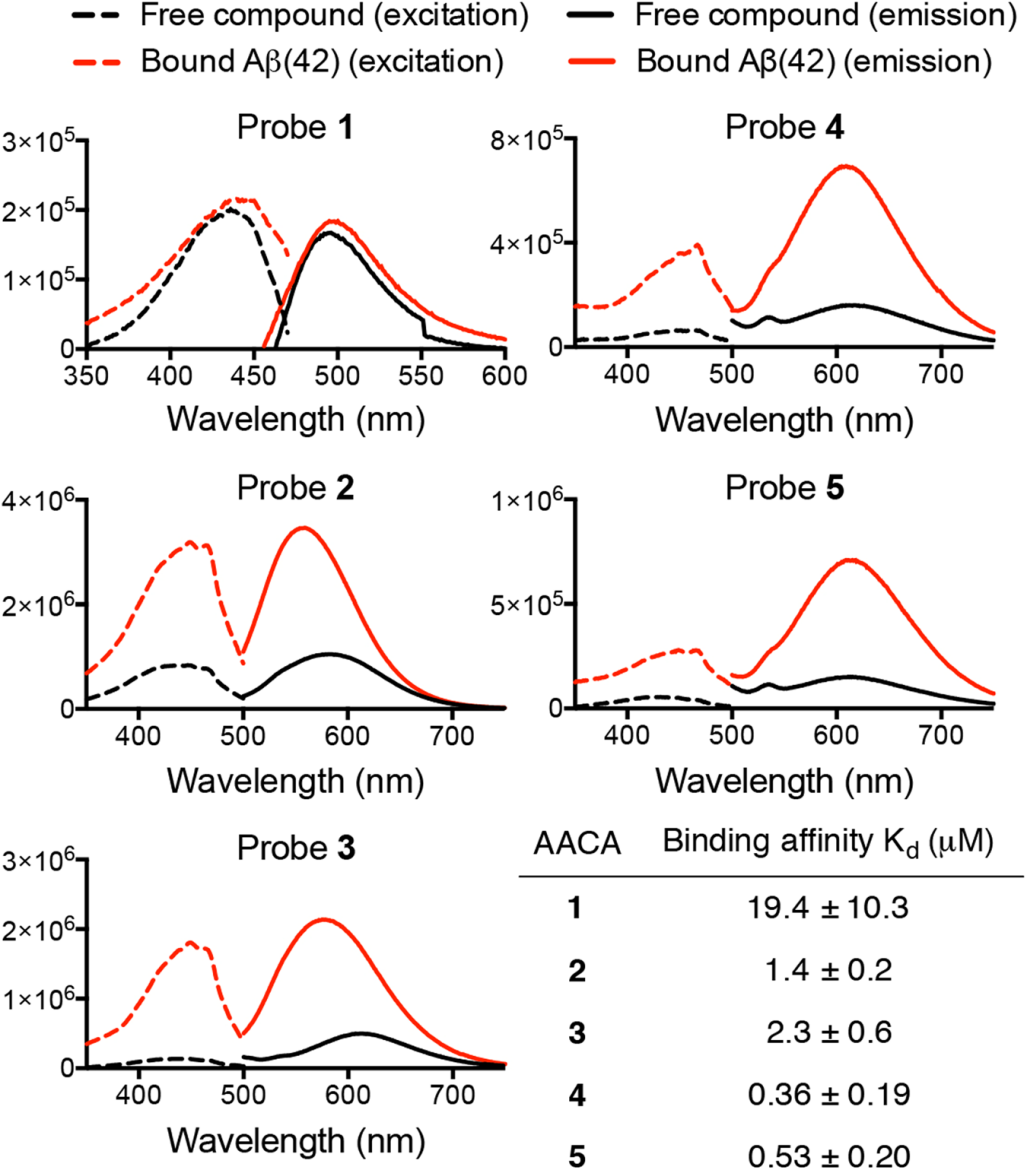
Fluorescence binding profiles and binding affinities of AACA probes **1–5** free (black) and bound (red) to β-amyloid (1–42) fibrils in solution.

In order to investigate whether the AACA probes **1–5** could serve as diagnostic tools for amyloid deposition in tissue, we examined the cellular toxicity of the probes in SH-SY5Y neuroblastoma cells. After 24 hours of dosing, we did not observe toxicity at concentrations up to 200 µM for any of the probes compared to control cells (Supplementary Figure 3). Next, we measured the binding and fluorescence properties of these compounds in the presence of fully aggregated Aβ(1–42) peptides in solution (Fig. 5)^31–33,52^. All probes **1–5** were found to bind to aggregated Aβ with affinities in the low micromolar to high nanomolar range (see Supplementary Figure 4a–d for binding curves). Probe **1** had the weakest binding affinity of all probes examined and exhibited the smallest enhancement of absorbance and fluorescence emission in the presence of aggregated Aβ. Interestingly, probes **4** and **5** did not exhibit a hypsochromic shift in fluorescence emission upon binding Aβ aggregates compared to probes **2** and **3**, suggesting minimal interactions between the fluorogenic cores of these probes and amino acid residues within the amyloid binding pockets. Probes **2** and **3**, on the other hand, had similar binding affinities and both exhibited large hypsochromic shifts for fluorescence emission upon binding to aggregated Aβ. Furthermore, we also observed an expected decrease in fluorescence emission intensity and quantum yield upon binding of heterocyclic aromatic compounds **3–5** compared to the fluorescence emission intensity of probe **2** (Supplementary Figure 4e–f), which can be attributed to increased modes of non-radiative relaxation of excited states due to the presence of the extended conjugated π systems^53,54^.

Although this work focuses on examination of the fluorescence properties of AACA probes when bound specifically to amyloid fibrils, we were interested in assessing whether the fluorescence emission of AACA probes was also sensitive to different aggregation states (e.g., oligomers versus fibrils) of amyloidogenic proteins and peptides. In an initial study towards this goal, we did not observe a significant shift in the fluorescence emission λ_max_ for probe **2** when this probe was exposed to Aβ(1–42) peptides at various time points during the aggregation process, which contained different mixtures of soluble aggregates (oligomers, protofibrils, and fibrils) that changed over the course of aggregation (see Supplementary Figure 5a–c). An increase in the maximum fluorescence intensity, however, was observed to correlate with increasing abundance of large aggregates (see Supplementary Figure 5d).

**Figure 6:**
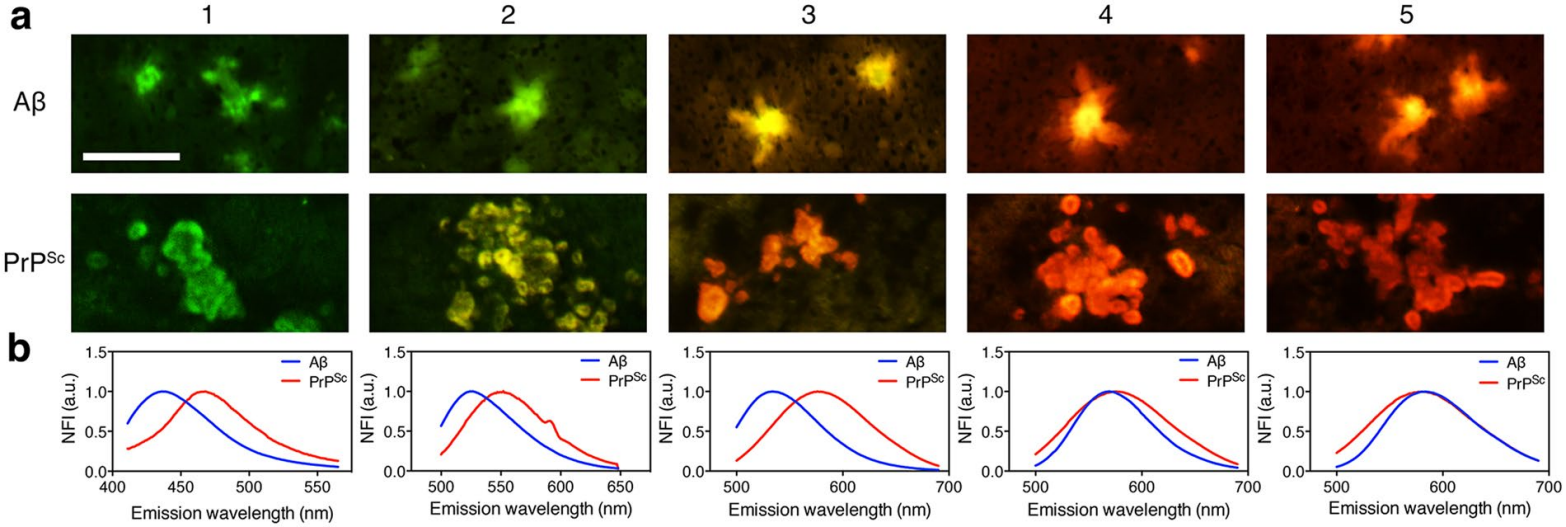
Brain tissue staining with AACA probes **1–5**. (**a**) Real-color fluorescence images of probes bound to Aβ plaques in the hippocampus of transgenic AD mice or to prion (PrP^SC^) plaques in the corpus callosum of prion-infected mice. (**b**) Spectroscopic measurements of the fluorophore emission of representative plaques stained with probes **1–5**. Scale bar = 50 microns.

To evaluate whether probes **1–5** could discriminate amyloids of different disease origin in brain samples, we incubated brain tissue slices from a transgenic (Tg) mouse model for Alzheimer’s disease (19959 PRNP-APP_swe/Ind_) and from mice inoculated with infectious mouse-adapted chronic wasting disease (mCWD) prions with each probe **1–5** (see supporting information for experimental details of these staining and microscopy protocols)^55–57^. True-color microscopy of the resulting stained tissues revealed that each compound bound specifically to amyloid deposits from both types of brain samples (Fig. 6a). Probes **1–3** exhibited fluorescence emission colors that were spectroscopically distinct for the different proteins related to the different diseases (from inspection of an average of 4–20 plaques per tissue slice). The emission profiles for probes **1–3** bound to Aβ diffuse and dense-core deposits in the hippocampus of Tg AD mice were blue-shifted compared to the same probes bound to prion aggregates in the corpus callosum of the prion-inoculated mice. Emission spectra of the stained amyloids (Fig. 6b and Supplementary Figure 6) revealed discrete differences in emission wavelengths that were characteristic of amyloid composition, with a general indication from probes **1–3** that prions contained binding pockets for the fluorophores that were more polar than the binding pockets on Aβ plaques. Consistent with the solution-based studies of the environmental sensitivity of probes **1–5** (Fig. 3), probe **3** exhibited the greatest capability to discriminate between amyloid deposits associated with prion or Alzheimer’s disease, displaying the largest difference in maximal emission wavelength of 41 ± 9 nm when bound to Aβ or prion plaques. Interestingly, probes **4** and **5** could not spectroscopically discriminate Aβ versus prion deposits in tissue (Fig. 6), which was again consistent with the hypothesis that there may be minimal interaction between the fluorogenic cores of probes **4** and **5** and the amino acid side chains lining the molecular binding pockets within the different amyloids.

Closer inspection of dense-core plaques stained with probes **3**, **4** and **5** suggested that, while two of the three probes were unable to discriminate between amyloid deposits of different protein origin, all three of these probes exhibited slightly different emission profiles for the core of the plaques compared to the peripheral dystrophic neurites radiating from the core (see Supplementary Figure 6)^58,59^. These findings suggest that these probes may also exhibit some sensitivity to different aggregation states of amyloids with the same (or similar) protein composition, thereby complementing the protein identity-distinguishing capabilities of probes **1–3**^22,60^.

### Discrimination of Amyloid Aggregates Using a Fluorescence Fingerprint Assay

**Figure 7:**
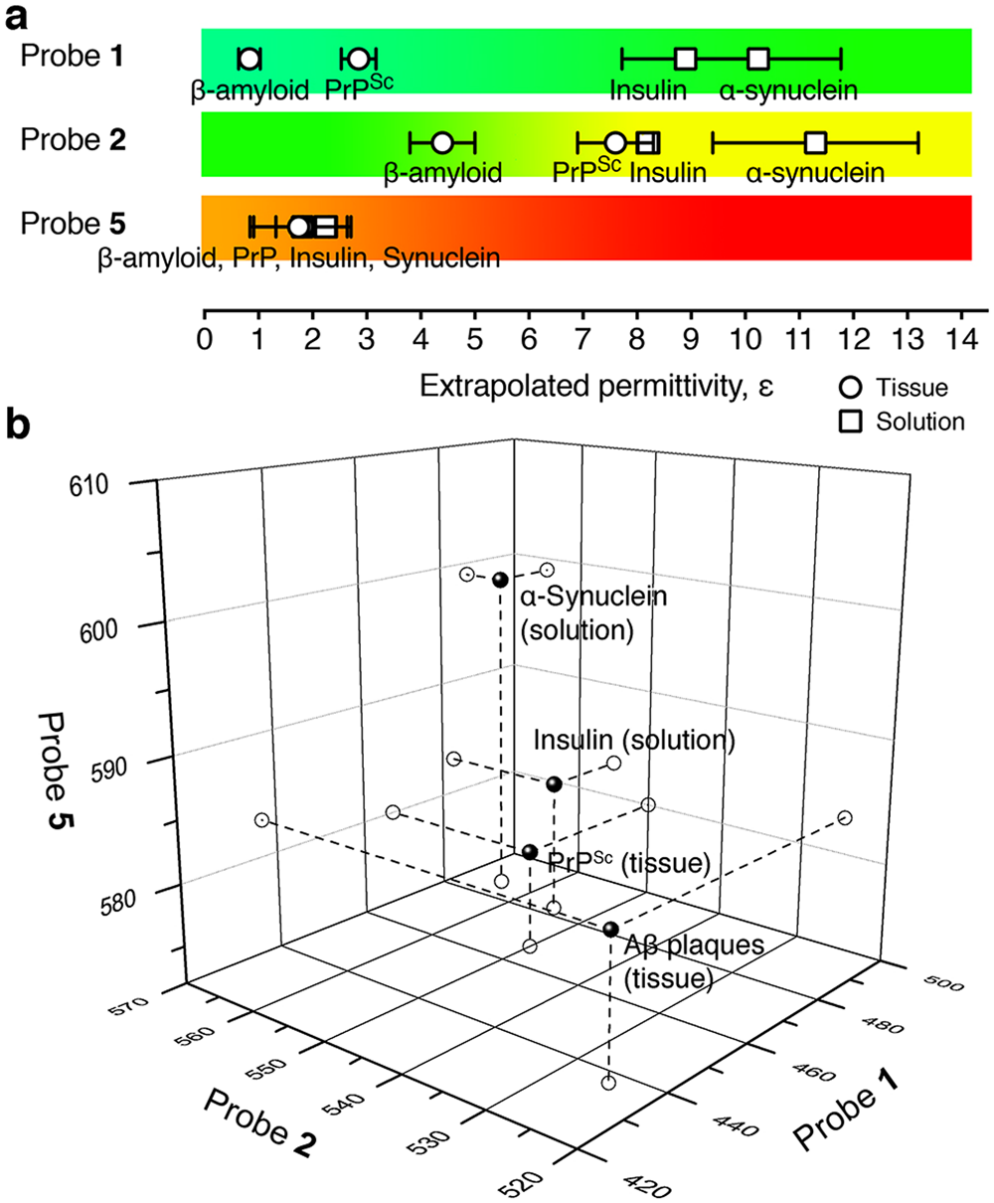
Discrimination of amyloid aggregates using a fluorescence fingerprint assay. (**a**) Observed color of fluorescence emission and corresponding extrapolated permittivities of molecular binding pockets on 4 different amyloids using AACA probes **1**, **2**, and **5**. (**b**) A three-dimensional fluorescence fingerprint analysis showing distinct 3 dimensional spectroscopic signatures (black spheres) for each protein aggregate using all 3 probes. The axes labels represent fluorescence emission wavelength in nanometers.

Despite the capability of probes **1–3** to fluorescently discriminate Aβ or prion deposits in neural tissue, Fig. 1 shows that many amyloids contain molecular binding pockets with similar polarities that make them difficult to identify conclusively by simple inspection of fluorescence of a single amyloid-bound AACA probe. We, therefore, examined whether using multiple probes could offer additional advantages to spectroscopically identify amyloids through a multi-dimensional fingerprint analysis (Fig. 7)^61,62^.

To demonstrate proof-of-concept, we generated calibration curves (see Supplementary Figure 7) for the fluorescence emission of probes **1**, **2**, and **5** in various solvents and evaluated their fluorescence properties when bound to Aβ or prion deposits in tissue and insulin or α-synuclein aggregates in solution^63^. Figure 7a shows that none of these probes alone could unambiguously spectroscopically distinguish all 4 of these amyloids from each other. However, when we generated a 3-dimensional plot of the fluorescence emission of these 3 probes bound to the 4 different amyloids, we found that each amyloid produced a distinct spectroscopic signature that could be used for conclusive identification of the major protein component in the amyloid (Fig. 7b).

## Conclusions

We have, thus, demonstrated that environmentally sensitive amyloid-binding compounds can be used to spectroscopically differentiate different amyloid species based on protein binding pocket composition. The solvatochromic properties of these probes can reveal small differences in the polar microenvironments within the binding pockets on amyloids, which are generally quite hydrophobic. We used the Lippert-Mataga model, which considers inherent parameters such as the excited and ground state dipole moments and the Onsager radii of fluorophores, for initial design of new environmentally sensitive fluorophores that are capable of discriminating between different amyloid aggregates in solution or in tissue. However, *ab initio* solvation studies reveal that restricted rotational degrees of freedom on one fluorophore may enhance environmental sensitivity, suggesting that the conformational dynamics of the fluorophores should also be considered in the design of amyloid discriminating probes.

While this work demonstrates that the relative permittivity of the binding pocket of an amyloid is an intrinsic property that can potentially be exploited for identification of the amyloidogenic protein, in many instances the permittivities (which reflect the polar microenvironment within the binding pockets of amyloids) are similar between amyloids of different composition and disease origin. This challenge of identifying the protein content within each amyloid can be overcome by using a fluorescence fingerprint assay, where using multiple AACA probes offers additional capabilities to spectroscopically differentiate such amyloids with similar permittivities. Recent results from a phase II clinical study has already demonstrated that AACA probe **2** (also known as Aftobetin-HCl) can be used to identify living Alzheimer’s patients by fluorescently labeling amyloid-containing deposits in the eyes^64^, demonstrating one possible translational opportunity for fluorescent amyloid-binding probes in clinical diagnosis. Fluorophores that can discriminate amyloids and identify disease origin may provide additional advantages for such novel optical platforms for diagnosis of specific amyloid-based diseases^36,40,44,64–66^.

## Methods

### General chemical methods and instrumentation for analysis

Unless otherwise indicated, all commercially available chemical reagents and anhydrous solvents were purchased at the highest commercial quality and were used as received without further purification. All non-aqueous reactions were carried out under argon atmosphere using dry glassware that had been flame-dried under a stream of argon unless otherwise noted. Anhydrous tetrahydrofuran (THF) and dichloromethane (CH_2_Cl_2_) were obtained by passing commercially available pre-dried, oxygen-free formulations through activated alumina columns. Flash column chromatography was performed on silica gel (Merck Kiesel gel 60, 230–400 mesh) using Hexanes-EtOAc or EtOAc-MeOH or toluene-acetone or diethyl ether mixtures of increasing polarity. The progress of all the reactions was monitored by thin-layer chromatography (TLC) using glass plates precoated with silica gel-60 F254 to a thickness of 0.5 mm (Merck), and compounds were visualized by irradiation with UV light and/or by treatment with a solution of CAM stain or potassium permanganate (KMnO_4_) in water stain followed by heating. ^13^C NMR and ^1^H NMR spectra were recorded on either 400 MHz or 500 MHz Varian instrument or 500 MHz JEOL instrument. CDCl_3_ was treated with anhydrous K_2_CO_3_, chemical shifts (δ) are quoted in parts per million (ppm) referenced to the appropriate residual solvent peak reference (CDCl_3_), with the abbreviations s, br s, d, t, q, m, td, dt and qd denoting singlet, broad singlet, doublet, triplet, quartet, multiplet, quartet of doublets, triplet of doublets, doublet of triplets and quartet of doublets respectively. J = coupling constants given in Hertz (Hz). High-resolution Mass spectra (HRMS) were recorded on a trisector WG AutoSpecQ spectrometer. Solvent permittivity values (dielectric constants and refractive index values) for solvatochromism fluorescence assays were obtained from Sigma and CRC Press^67,68^.

### Chemical synthesis

The supplementary information describes the details for the synthesis of AACA probes **1–5**.

### Biological samples

Tissue samples of mice brain tissue with β-amyloid (Aβ) plaques, prion (PrP^Sc^) deposits, and serum amyloid A (SAA) deposits, and human brain tissue containing Aβ senile plaques were generously provided by Professor C. Sigurdson and colleagues (UCSD School of Medicine). Human patient samples were obtained from the UCSD Alzheimer’s Disease Research Center. Institutional board review was obtained from the UCSD Human Research Protections Program in accordance with the Health Insurance Portability and Accountability Act. Synthetic β-amyloid (1–42) was purchased from PL Laboratories (Port Moody, British Columbia) as a lyophilized powder and stored in an anhydrous environment at −80 °C until use. Full length recombinant α-synuclein was generously provided by Professor D. Selkoe (Harvard Medical School) as a frozen stock in 1xPBS, stored at −80 °C. Recombinant human insulin was purchased from Sigma Aldrich (no. 11376497001, United States). Fibrillized amyloid aggregates of the seminal peptides SEVI (also known as PAP(248–286) or semen derived enhancer of viral infection), PAP(85–120), and semenogelin-1 (SEM-1(49–107)), and the bacterial peptide fragments protegrin-1 (PG-1), and curli fragments CsgA-R1 and CsgA-R5 were graciously provided by Professor S. Dewhurst and coworkers from the University of Rochester Medical Center. Samples were stored frozen in 1xPBS or deionized H_2_O at −80 °C.

### Probe 2 calibration curve and extrapolation of binding pocket permittivities

A Lippert-Mataga calibration plot that correlates the observed fluorescence emission to the full Lippert-Mataga parameters or the dielectric parameter alone was generated in accordance to equation (S1) and equation (2), respectively (Supplementary Figure 1)^32,33^. In the spectroscopic ruler measurements for various amyloid species using probe **2**, we utilized a calibration curve that includes protic and aprotic solvents; the Lippert-Mataga calibrations developed for the solvatochromism assay to study chemical modifications to the fluorophore scaffold were obtained in aprotic solvents only in order to avoid the potential influence of intermolecular forces such as Hydrogen-bonding between protic solvent molecules and the probes that could mask the effect of solvent permittivities on the spectroscopic properties of the probes. Brain tissue plaques of Aβ, prions (PrP^Sc^), and serum amyloid A (SAA), and fibrillized aggregates of insulin, Aβ(1–42), α-synuclein, protegrin-1 (PG-1), curli (CsgA-R1 and CsgA-R5), semen derived enhancer of viral infection (SEVI, or PAP(248–286)) and related fragment (PAP(85–150), and SEM-1 (semenogelin-1) were then stained or bound with ANCA probe **2**^33^. Fluorescence emission wavelengths at the maximum intensity, λ_max_, were converted to permittivities using equation (2).

### Solvatochromism studies of AACA probes 1–5

Each probe was dissolved in freshly distilled anhydrous acetonitrile (ACN), acetone, dichloromethane (DCM), tetrahydrofuran (THF), diethyl ether (Et_2_O), anisole, and toluene to a final concentration of 1.5 μM. Excitation and fluorescence emission spectra of each solution were obtained on a PTI spectrofluorimeter under argon in 1 nm increments from 350–520 nm and 400–650 nm, respectively. The wavelength corresponding to the maximum intensity was taken as λ_max_. For absorption studies, compound solutions in anhydrous solvents under argon were prepared at 5 μM. Absorption spectra were obtained from a NanoDrop 2000 UV-Vis spectrophotometer (ThermoFisher, cat. ND-2000) using a clear quartz cuvette. Because equation (2) serves as an empirical description for the dependence of fluorescence emission of AACA probes **1–5** on the local dielectric environment, Lippert-Mataga plots were graphed showing correlation of the emission, absorption, and Stokes Shift bands to the solvent permittivity (*e*.*g*. the dielectric constant with and without the refractive index contribution, Supplementary Figure 2).

### Computational studies of AACA probes

All Density Functional Theory (DFT) calculations were carried out with Gaussian 09 (revision D.01) electronic structure package with a 10^−8^ convergence criterion for the density matrix elements, using the hybrid-meta GGA functional M062X^69,70^. The fully optimized contracted triple-ζ all electron Gaussian basis set developed by Ahlrichs and co-workers was employed for all the elements^71^. The studied systems have been fully optimized in both ground and excited states. For the excited state optimization, the UV-Vis spectrum was calculated within the Time-Dependent Density Functional Theory (TDDFT) methodology using the same functional as for the optimizations^72^. The number of excited states included in the TDDFT calculations is 20. We performed TDDFT geometry optimizations in order to find the minimum energy point on the excited state potential energy surface in all cases (vacuum and solvents) using the HOMO-LUMO excitation peak. The SMD solvation model from Truhlar and coworkers was used to model the different studied solvents.

### MTT Cell Proliferation Assay in SH-SY5Y Neuroblastoma

SH-SY5Y cells (ATCC # CRL-2266) were cultured in 1:1 Eagle’s Minimum Essential Medium (EMEM): F12 medium supplemented with 10% FBS and maintained at 37 °C in a humidified atmosphere with 5% CO_2_. An MTT cell proliferation assay was performed as previously reported with noted changes^73^. Briefly, SH-SY5Y cells were plated in 96-well dishes at a density of 30,000 cells/well in 100 µL of EMEM/F12 supplemented with 10% FBS. After adhering overnight, cells were dosed with various concentrations of AACA probes **1–5**, with final concentrations ranging from 0–200 µM (1% DMSO) for 24 hours at 37 °C. After 24 hours of incubation, 20 μL of the MTT reagent (Biotium) was added to each well and the cells were let incubate at 37 °C for 2 additional hours. All solutions were then removed and the formazan crystals were subsequently solubilized with DMSO (100 μL). The cell viability was determined by measuring the absorbance at 570 nm using a Molecular Devices SpectraMax i3 multimode plate reader. All results were expressed as percent reduction of MTT relative to control cells (defined as 100% viability) and the average absorbance value for each treatment was blanked with the absorbance reading of media control blanks. Each concentration was done in at least triplicate.

### Fluorescence studies of probes with aggregated Aβ(1–42) and other amyloidogenic proteins in solution

Aggregated Aβ peptide was prepared by dissolving Aβ (1–42) in 1xPBS pH 7.4 to a final concentration of 100 µM. This solution was stirred at 1200 rpm for 3 days at room temperature. The aggregation state was verified by a standard thioflavin T fluorescence assay^74^.

α-Synuclein aggregates were similarly prepared by dissolving α-synuclein in sterile 1xPBS pH 7.4 to a final concentration of 100 µM. Solutions were incubated at 37 °C for 7 days prior to use. The presence amyloid fibrils was verified by standard thioflavin T fluorescence assay.

Insulin amyloid aggregates were prepared using a modified version of the protocol described by Jiménez and coworkers^75^. Briefly, monomeric insulin was dissolved in pH 1.6 H_2_O at 65 °C for 8 days. Aggregates were then dialyzed into pH 7.4 1xPBS for 24 hours at 25 °C and subsequently incubated for 48 hours at 65 °C before storing at 37 °C prior to measurements. The presence of amyloid fibrils was verified by a standard thioflavin T fluorescence assay.

Tau was not included in the analysis due to the inability of ANCA compound **2** to stain neurofibrillary tangles (NFTs) in human brain samples from AD patients (data not shown).

A 15 μL of stock (100 μM) aggregated amyloid solutions were added to 285 μL of the AACA probes (in 5% DMSO in nanopure water) to attain a final concentration of 5 μM amyloid aggregate and 4 μM of the probe. Concentrations were chosen assuming a 1:1 probe to fibril binding stoichiometry to avoid oversaturation of the fluorimeter detector. The solutions were transferred to a 300 μL cuvette and the fluorescence was measured at 22.5 °C. The excitation wavelength varied depending on the maximal absorbance of the probe; AACA probe **1** was excited at 420 nm, probes **2–5** were excited at 450 nm.

### Time Course Aggregation Study of Aβ with ANCA Probe 2

Aβ(1–42) was initially solubilized in 100% HFIP to 1 mM concentration at RT for 24 hours with shaking. The solution was sonicated and vortexed before it was diluted in cold nanopure water (2:1 H_2_O: HFIP). Aliquoted fractions were lyophilized for 2 days, followed by storage at −80 °C until use. HFIP treated lyophilized Aβ was solubilized in DMSO (clear solution) to a final concentration of 1 mM. The solution was then diluted into PBS to 100 μM and centrifuged at 14,000 rpm for 10 minutes at 4 °C. The supernatant (t = 0) was taken and allowed to aggregate at 37 °C. At various time points (t = 0–120 hours) aliquots were removed to measure the fluorescence binding of probe **2** with Aβ and to be frozen down for gel analysis. The emission spectra (excitation at 450 nm) were taken using a final concentration of 4 uM of probe **2** with 5 uM Aβ in 5% DMSO/DI H_2_O (using fresh, anhydrous DMSO ampules) Gel analysis of Aβ was done under non-reducing SDS-PAGE and visualized with silver staining (Pierce Silver Stain Kit).

### Determination of binding constants to aggregated Aβ (1–42) peptides

As previously reported^31,32^, in order to estimate the binding constant (K_d_) for the probe-Aβ complexes from the fluorescence studies, we made the following assumptions: (1) All probes are completely in solution and free of any significant competing binding process such as self-aggregation. (2) The concentration of unbound probes can be approximated as close to the total concentration of the probes. (3) The binding sites in the aggregated Aβ peptides are not completely occupied at the concentration of Aβ binding probes used for the fluorescence studies (i.e., the experiments are carried out under non-saturated binding conditions).

Pre-aggregated Aβ(1–42) (5 μM final concentration) was mixed with various concentrations of probes **1–5** in 5% DMSO in nanopure water and their fluorescence emission recorded. Samples were excited at 420 nm for probe **1**, and 450 nm for probes **2–5**. The K_d_ values were determined by fitting data to a one-site specific Hill-binding model (Supplementary Figure 3).

### Histology staining of mouse brain samples with AACA probes 1–5

Male and female transgenic mice overexpressing wild type mouse PrP (Tga20) were inoculated with the mouse-adapted prion strain mCWD and were euthanized upon developing terminal signs of prion disease. Transgenic mice (19959) harboring the Aβ plaques express the mutant human amyloid precursor protein APP_SweInd_, which bears both the Swedish (K670N/M671L) and the Indiana (V717F) mutations, under the control of the Syrian hamster prion protein promoter. Mice were euthanized between 9–13 months of age and their brains extracted into optical cutting temperature (OCT) fixation media. Frozen brain sections were cut from these frozen blocks.

Frozen tissue sections were dried for 1 hr, hydrated with 100%, 95%, and 70% ethanol for 5 min each, and then rinsed with deionized water. Sections were then buffered with phosphate-buffered saline (1X PBS) for 30 min. Probes **1–5** were diluted 1:50 in 1X PBS (from stock solutions of 3 mM) to a final concentration of 60 μM, added to brain sections, incubated for 30 min at room temperature, washed three times with 1X PBS, and coverslipped using DAKO fluorescent mounting media.

In order to obtain spectral scans of probes bound to amyloid plaques in neural tissue slices, brain samples were excited with a 405 nm (probe **1**) or 488 nm (probes **2**–5) laser on an Olympus FluoView FV1000 spectral deconvolution confocal microscope with 5% power under 10X magnification. The emission spectra of probes **1–5** bound to Aβ, PrP^Sc^, and background were collected in 1 nm increments from 450–650 nm. A minimum of 10 measurements was collected for each compound bound to plaque and non-plaque regions as a background control. The wavelength corresponding to the maximum relative fluorescence intensity was taken as the emission λ_max_. Real color brain sections were imaged under an Olympus MVX10 Macroview equipped with a MWB2 (Japan) long-pass filter and a 2X MVX (PF) Plan Apochromat lens with a 0.5 numerical aperture. Each sample was illuminated with epifluorescence and imaged with an exposure time of 0.2–0.8 sec, depending on the sample brightness.

### Lippert-Mataga calibration plots for AACA probes 1–5

The calibration curves based on the reduced Lippert-Mataga solvatochromism (equation (2)) were used to extrapolate binding pocket permittivities from measured fluorescence emission values (Supplementary Figure 7). The slopes (*m*) of the best-fit linear regression lines for equation (2) (y = *m · x* + *b*) were used to extract the binding pocket permittivity (*x*) from the observed fluorescence emission λ_max_ (*y*). Slope values for all probes are tabulated in Supplementary Table 2.

### Data Availability

All data generated or analyzed during this study are included in this published article (and its Supplementary Information files).

## Electronic supplementary material

Supplementary Information
