# Supplementary material for: Solvation-Guided Design of Fluorescent Probes for Discrimination of Amyloids
Source: Sci Rep. 2018 May 3;8:6950. doi: 10.1038/s41598-018-25131-2 (PMC5934448; doi:10.1038/s41598-018-25131-2)
Supplement: Supplementary file 1 — Supplementary Information [file 41598_2018_25131_MOESM1_ESM.pdf]

Supplementary Information

Solvation-Guided Design of Fluorescent Probes for Discrimination of Amyloids

Kevin J. Cao[a], Kristyna M. Elbel[a], Jessica L. Cifelli[a], Jordi Cirera[a,†], Christina Sigurdson[b], Francesco Paesani[a], Emmanuel A. Theodorakis[a], and Jerry Yang[a,*]

Table of Contents

1. Chemical Synthesis S1
2. Simplification of the Lippert-Mataga equation S8
3. Supplementary Figure 1 .... S10
4. Supplementary Figure 2 .... S11
5. Supplementary Figure 3 .... S12
6. Supplementary Figure 4 .... S13
7. Supplementary Figure 5 .... S14
7. Supplementary Figure 6 .... S15
7. Supplementary Figure 7 .... S16
8. Supplementary Table 1 .... S17
9. Supplementary Table 2 .... S17
10. Supplementary Table 3 .... S17
11. Supplementary Table 4 .... S18
12. Supporting References S18
13. ^{1}H and ^{13}C NMR spectra of AACA probes **1, 3 – 5** S19

Chemical Synthesis

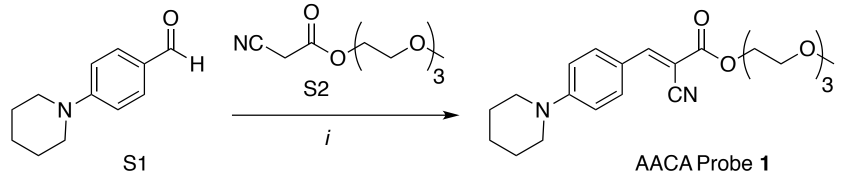

Synthesis of AACA Probe **1**. Conditions: *i.* DBU, THF, 60°C.

(E)-2-(2-(2-methoxyethoxy)ethoxy)ethyl 2-cyano-3-(4-(piperidin-1-yl)phenyl) acrylate (AACA Probe 1)

Commercially available 4-piperidinyl benzaldehyde (S1) (0.05 g, 0.255 mmol) was dissolved in THF (2.0 mL) under nitrogen. Previously reported 2(2-(2-methoxyethoxy)ethoxy)ethyl-2-cyanoacrylate (S2)[1,2] (0.216 mmol) and 1,8-diazabicyclo[5.4.0]undec-7-ene (DBU) (0.02 mmol) were then added and the mixture left stirring at 30°C. The reaction was monitored by TLC, revealing that the reaction was completed in 6 hrs. The crude solution was concentrated under reduced pressure and the product purified with flash chromatography (1:1 hexanes/ethyl acetate) to give probe **1** as an orange solid (90%). R_f =0.70 (1:1 hexanes/ethyl acetate);

^{1}H NMR (500 MHz, $CDCl_3$) δ 8.06 (s, 1H), 7.91-7.93 (d, J = 9.0 Hz, 2H), 6.84-6.86 (d, J = 9.5 Hz, 2H), 4.41-4.43 (m, 2H), 3.80-3.82 (m, 2H), 3.72-3.74 (m, 2H), 3.67-3.69 (m, 4H), 3.54-3.56 (m, 2H), 3.45-3.46 (m, 4H), 3.37 (s, 3H), 1.67-1.68 (m, 6H); ^{13}C NMR (125 MHz, $CDCl_3$) δ 164.3, 154.6, 154.2, 134.4, 120.2, 117.4, 113.4, 94.4, 72.1, 71.0, 70.8, 70.7, 69.1, 65.2, 59.2, 48.3, 25.5, 24.5; HRMS calcd. for $C_{22}H_{30}N_2O_5$ $[M+Na]^+$ 425.20, found 425.24

(E)-2-(2-(2-methoxyethoxy)ethoxy)ethyl 2-cyano-3-(6-(piperidin-1-yl)naphthalen-2-yl)acrylate (AACA Probe 2)

AACA Probe **2** was synthesized as previously described.[1]

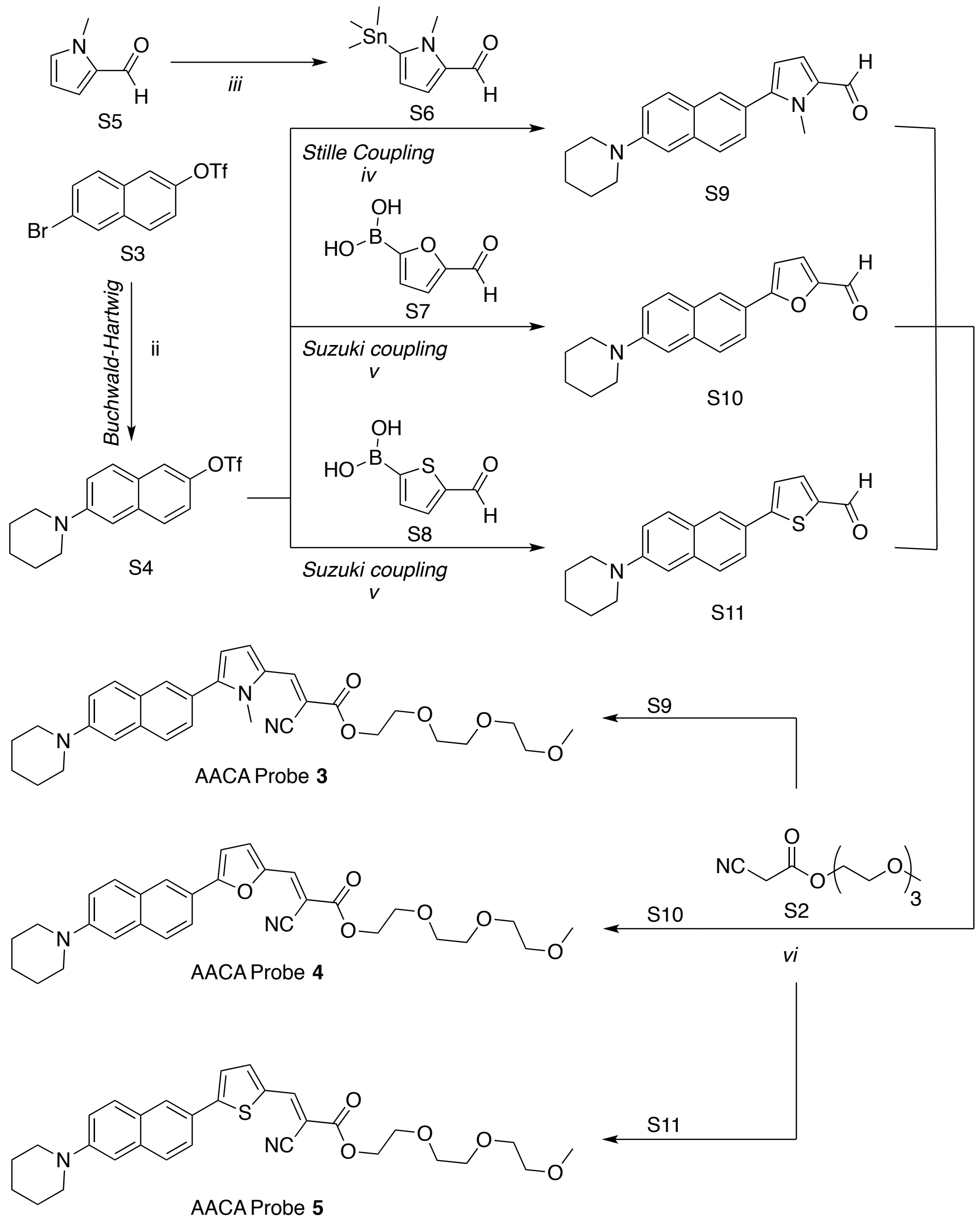

Synthesis of AACA probes **3 – 5**. Conditions: *ii.* $Pd(OAc)_2$, $P(tBu)_3$, NaO*t*Bu, piperidine, toluene, 110°C. *iii*. *N*-methyl piperazine, *n*BuLi, TMEDA, Me_3SnCl. *iv*. $PdCl_2(PPh_3)_2$, DMF. *v*. $Pd(PPh_3)_4$, K_2CO_3, THF. *vi.* DBU, THF, r.t. 5 minutes.

6-(piperidin-1-yl)naphthalen-2-yl trifluoromethanesulfonate (S4)

To a microwave vial containing degassed toluene (6.7 mL), commercially available 6-bromonaphthalen-2-yl trifluoromethanesulfonate (S3) (0.25 g, 0.704 mmol), $Pd(OAc)_2$ (0.008 g,

0.0352 mmol), and NaOtBu (0.088 g, 0.915 mmol) was added and allowed to stir for 5 minutes under argon at room temperature followed by addition of $P(t\text{-}Bu)_3$ (0.0704 mmol) via syringe and continued stirring for an additional 30 minutes. Piperidine (0.845 mmol) dissolved in toluene (0.845 mL) was added dropwise via syringe and the vial was sealed and heated to 110°C for 12 hours. The reaction mixture was then allowed to cool to room temperature and subsequently it was diluted with CH_2Cl_2 (5 mL), filtered through a pad of celite, and concentrated. The crude material was then adsorbed on to silica and chromatographed (0-20% EtOAc/hexanes) to give S4 as a white solid (0.152 mg, 60%). R_f = 0.59 (10 % EtOAc/hexanes)

^{1}H NMR (400 MHz, $CDCl_3$) δ 7.69-7.72 (d, J = 12.0 Hz, 2H), 7.59 (m, 1H), 7.35-7.38 (dd, J = 8.0 Hz, 4.0 Hz, 1H), 7.25-7.27 (m, 1H), 7.11 (bs, 1H), 3.29 (m, 4H), 1.73-1.79 110 (m, 4H), 1.62-1.67 (m, 2H); ^{13}C NMR (100 MHz, $CDCl_3$) δ 151.0, 145.2, 134.1, 128.7, 127.6, 121.5, 120.5, 119.8, 118.9, 117.4, 109.7, 50.6, 25.8, 24.4; HRMS calcd. For $C_{16}H_{17}F_3NO_3S$ $[M + H]^+$ 360.0876, found 360.0876

1-methyl-5-(trimethylstannyl)-1H-pyrrole-2-carbaldehyde (S6)

To a solution of N-methyl piperazine (0.535 g, 5.038 mmol) and TMEDA (1.17 g, 10.076 mmol) in hexanes (10 mL) was added *n*BuLi (3.4 mL, 5.038 mmol) at -40°C and allowed to stir for 30 minutes. Then 1-methylpyrrole carbaldehyde (S5) (0.5g, 4.58 mmol) was added dropwise at -40°C and reaction continued to stir for an additional 45 minutes. A second portion of *n*BuLi (6.3 mL, 10.076 mmol) was added dropwise at -40°C and the reaction was allowed to stir for 1 hour. Trimethyl tin chloride solution (1.0M, 10.076 mmol) was added dropwise and the reaction was allowed to come to room temperature and stir overnight. The reaction was then quenched with water, extracted with diethyl ether (3 x 10 mL), dried with anhydrous $MgSO_4$, and concentrated in

vacuo to give S6 (442 mg, 35%) as a clear, pale yellow oil which was used without further purification. R_f = 0.71 (20 % EtOAc/hexanes)

^{1}H NMR (500 MHz, $CDCl_3$) δ 9.53 (s, 1H), 6.93 (d, J = 4.0 Hz, 1H), 6.29 (d, J = 4.0 Hz, 1H), 3.99 (s, 3H), 0.38 (s, 9H); ^{13}C NMR (125 MHz, $CDCl_3$) δ 178.5, 148.1, 135.5, 124.4, 119.1, 37.5, 18.8; HRMS calcd. For $C_9H_{16}NOSn$ $[M+H]^+$ 274.0250, found 274.0247

1-methyl-5-(6-(piperidin-1-yl)naphthalen-2-yl)-1H-pyrrole-2-carbaldehyde (S9)

To a solution of S6 (0.05 g, 0.184 mmol) and S4 (0.0794 g, 0.221 mmol) in degassed DMF (0.2 mL) under argon was added $PdCl_2(PPh_3)_2$ (0.0092 mmol) and the reaction was heated to 60°C and allowed to stir for 30 minutes. The reaction was then cooled to room temperature and diluted with water (0.2 mL) and diethyl ether (0.2 mL) and filtered through a pad of celite. The reaction mixture was the extracted with diethyl ether (3 x 1 mL) and combined organic layers were washed with brine (1 x 2 mL), dried with anhydrous $MgSO_4$, and concentrated. The residue was adsorbed on to silica and purified by flash column chromatography (0-5% EtOAc/hexanes) to give S9 (42 mg, 72%) as a light yellow powder. R_f = 0.46 (20 % EtOAc/hexanes)

^{1}H NMR (400 MHz, $CDCl_3$) δ 9.58 (s, 1H), 7.73-7.75 (m, 3H), 7.41-7.43 (dd, J = 8.4 Hz, 2.0 Hz, 1H), 7.32- 7.35 (dd, J = 9.2 Hz, 2.4 Hz, 1H), 7.14 (d, J = 2.0 Hz, 1H), 7.00 (d, J = 4.4 Hz, 1H), 3.99 (s, 3H), 3.29-3.32 (m, 4H), 1.74-1.80 (m, 4H), 1.64-1.66 (m, 2H); ^{13}C NMR (100 MHz, $CDCl_3$) δ 179.5, 150.8, 145.1, 134.6, 133.0, 129.0, 128.3, 127.7, 127.0, 126.9, 125.4, 124.8, 120.7, 110.9, 109.8, 50.7, 34.7, 25.9, 24.5; HRMS calcd. for $C_{21}H_{23}N_2O$ $[M+H]^+$ 319.1805, found 319.1809

5-(6-(piperidin-1-yl)naphthalen-2-yl)furan-2-carbaldehyde (S10)

6-(piperidin-1-yl)naphthalen-2-yl trifluoromethanesulfonate (S4) (50.0 mg, 0.139 mmol), (5-formylfuran-2-yl)boronic acid (S7) (44.0 mg, 0.209 mmol), K_2CO_3 (33.0 mg, 0.236 mmol), and $Pd(PPh_3)_4$ (24.0 mg, 0.0209 mmol) was dissolved in a mixture of THF (0.3 mL) and water (0.13 mL) in a microwave vial equipped with a stir bar and capped with a septum. The reaction mixture was degassed with argon, sealed, and microwaved for 1 hour at 50°C. The reaction mixture was then dried with anhydrous Na_2SO_4, condensed under reduced pressure and purified by silica flash chromatography (0-10% EtOAc/hexanes) to give S10 (38.4nmg, 91%) as a yellow solid. $R_f = 0.25$ (20 % EtOAc/hexanes)

^{1}H NMR (400 MHz, $CDCl_3$) δ 9.63 (s, 1H), 8.21 (s, 1H), 7.67-7.76 (m, 3H), 7.26-7.34 (m, 2H), 7.08 (d, J = 2.0 Hz, 1H), 6.85 (d, J = 3.6 Hz, 1H), 3.31 (m, 4H), 1.64-1.77 (m, 6H); ^{13}C NMR (100 MHz, $CDCl_3$) δ 177.0, 160.5, 151.8, 151.0, 135.5, 129.5, 127.6, 127.4, 123.0, 120.4, 109.6, 107.2, 50.4, 25.8, 24.5; HRMS calcd. for $C_{20}H_{20}NO_2$ $[M+H]^+$ 306.1489, found 306.1491

5-(6-(piperidin-1-yl)naphthalen-2-yl)thiophene-2-carbaldehyde (S11)

To a solution of 6-(piperidin-1-yl)naphthalen-2-yl trifluoromethanesulfonate S4 (0.01 g, 0.028 mmol) and (5-formylthiophen-2-yl)boronic acid (S8) (0.042 mmol) in a mixture of THF (0.06 mL) and water (0.025 mL) was added K_2CO_3 (0.0476 mmol) and $Pd(PPh_3)_4$ (0.0042 mmol). The solution was then degassed with argon and microwaved at 50°C for 1 hour. The reaction was then filtered through cotton and concentrated. The crude mixture was adsorbed on to silica and chromatographed (0-10% EtOAc/hexanes) to give S11 (5.9 mg, 66%) as a yellow powder. $R_f = 0.44$ (20 % EtOAc/hexanes)

^{1}H NMR (500 MHz, $CDCl_3$) δ 9.89 (s, 1H), 8.01 (s, 1H), 7.73-7.76 (m, 2H), 7.66-7.70 (m, 2H), 7.46 (d, J = 5.0 Hz, 1H), 7.32 (d, J = 10.0 Hz, 1H), 7.09 (bs, 1H), 3.31-3.33 (m, 4H), 1.76 (m,

4H), 1.65-1.67 (m, 2H); ^{13}C NMR (125 MHz, $CDCl_3$) δ 182.8, 155.4, 150.9, 141.7, 137.8, 135.3, 129.3, 127.8, 127.6, 127.5, 125.4, 124.4, 123.5, 120.6, 109.6, 50.4, 29.8, 25.8, 24.4; HRMS calcd. for $C_{20}H_{19}NOS$ $[M+H]^+$ 322.1260, found 322.1257

(E)-2-(2-(2-methoxyethoxy)ethoxy)ethyl 2-cyano-3-(1-methyl-5-(6-(piperidin-1-yl)naphthalen-2-yl)-1H-pyrrol-2-yl)acrylate (AACA Probe 3)

To a solution of S9 (0.02 g, 0.063 mmol) and S2 (0.0116 g, 0.05 mmol) in THF (0.25 mL) under argon was added DBU (0.001 mmol) and the reaction was allowed to stir at room temperature for 10 minutes. The reaction was then concentrated in vacuo, adsorbed on to silica, and purified by flash chromatography (5-80% EtOAc/hexanes) to give probe **3** (24.5 mg, 92%) as an orange viscous oil. R_f = 0.38 (80 % EtOAc/hexanes)

^{1}H NMR (500 MHz, $CDCl_3$) δ 8.16 (s, 1H), 7.87 (d, J = 4.0 Hz, 1H), 7.72-7.74 (m, 3H), 7.38-7.40 (dd, J = 8.5 Hz, 1.5 Hz, 1H), 7.33-7.35 (dd, J = 9.0 Hz, 2.0 Hz, 1H), 7.13 (bs, 1H), 6.55 (d, J = 4.5 Hz, 1H), 4.42-4.44 (m, 2H), 3.81-3.83 (m, 2H), 3.78 (s, 3H), 3.73-3.75 (m, 2H), 3.66-3.70 (m, 4H), 3.38 (s, 3H), 3.30-3.33 (m, 4H), 1.74-1.78 (m, 4H), 1.64-1.65 (m, 2H); ^{13}C NMR (125 MHz, $CDCl_3$) δ 164.7, 151.0, 145.2, 139.7, 134.7, 129.0, 128.4, 127.6, 127.2, 126.8, 125.2, 120.7, 120.6, 117.7, 114.0, 109.6, 91.1, 72.1, 71.0, 70.8, 70.7, 69.0, 65.2, 59.2, 50.6, 32.2, 29.8, 25.8, 24.4; HRMS calcd. for $C_{31}H_{37}N_3O_5Na$ $[M+Na]^+$ 554.2625, found 554.2626

(E)-2-(2-(2-methoxyethoxy)ethoxy)ethyl 2-cyano-3-(5-(6-(piperidin-1-yl)naphthalen-2-yl)furan-2-yl)acrylate (AACA Probe 4)

S10 (0.017 g, 0.0557 mmol) and S2 (0.0121g, 0.0524 mmol) were dissolved in THF (0.25 mL) under argon atmosphere. DBU (0.000557 mmol) was added via syringe and the reaction was

allowed to stir at room temperature for 5 minutes. The reaction was then concentrated, adsorbed on to silica, and purified via flash chromatography (10-90% EtOAc/hexanes) to give probe **4** (23.3 mg, 86%) as a red viscous oil. R_f = 0.3 (60 % EtOAc/hexanes)

^{1}H NMR (400 MHz, $CDCl_3$) δ 8.24 (s, 1H), 7.97 (s, 1H), 7.93 (m, 2H), 7.69-7.71 (m, 1H), 7.33 (m, 2H), 7.10 (bs, 1H), 6.94 (d, J = 4.0 Hz, 1H), 4.46 (m, 2H), 3.82-3.84 (m, 2H), 3.73-3.76 (m, 2H), 3.66-3.69 (m, 4H), 3.55-3.58 (m, 2H), 3.38 (s, 3H), 3.32-3.35 (m, 4H), 1.77 (m, 4H), 1.65 (m, 2H); ^{13}C NMR (100 MHz, $CDCl_3$) δ 163.5, 161.2, 151.0, 147.7, 138.2, 135.6, 129.9, 127.6, 125.9, 125.2, 123.0, 120.2, 116.3, 109.6, 108.9, 72.1, 71.0, 70.8, 70.7, 69.0, 65.5, 59.2, 59.2, 50.4, 29.8, 25.7, 24.4; HRMS calcd. for $C_{30}H_{34}N_2O_6Na$ $[M+Na]^+$ 541.2309, found 541.2310

(E)-2-(2-(2-methoxyethoxy)ethoxy)ethyl-2-cyano-3-(5-(6-(piperidin-1-yl)naphthalen-2-yl)thiophen-2-yl)acrylate (AACA Probe 5)

Compound S11 (0.05 g, 0.156 mmol) and S2 (0.125 mmol) were dissolved in THF (0.62 mL) under an argon atmosphere. DBU (0.0078 mmol) was added and the reaction was allowed to stir at room temperature for 5 minutes during which the reaction developed a dark red color. The reaction was then concentrated, adsorbed on to silica, and chromatographed (0-5% acetone/toluene) to give probe **5** (48.0 mg, 71%) as a red viscous oil. R_f = 0.38 (70 % EtOAc/hexanes)

^{1}H NMR (500 MHz, $CDCl_3$) δ 8.31 (s, 1H), 8.05 (bs, 1H), 7.75 (d, J = 5.0 Hz, 2H), 7.69 (bs, 2H), 7.47 (d, J = 5.0 Hz, 1H), 7.33 (bs, 1H), 7.08 (bs, 1H), 4.45-4.47 (m, 2H), 3.82 (m, 2H), 3.72-3.74 (m, 2H), 3.67-3.69 (m, 4H), 3.56 (m, 2H), 3.38 (s, 3H), 3.33 (m, 4H), 1.65-1.76 (m, 6H); ^{13}C NMR (125 MHz, $CDCl_3$) δ 174.1, 163.1, 156.1, 150.8, 146.9, 139.9, 134.2, 129.3, 127.7, 127.5, 127.0, 125.6, 124.2, 123.7, 120.4, 116.2, 109.6, 96.7, 77.1, 72.0, 70.9, 70.7, 70.6, 68.9, 65.4, 59.1,

50.3, 29.8, 25.7, 24.4; HRMS calcd. For $C_{30}H_{34}N_2O_5SNa$ $[M+Na]^+$ 557.2081, found 557.2079

Supplementary Methods:

Simplification of the Lippert-Mataga equation to a linear one-variable model

$$\tilde{\nu}_{absorption} - \tilde{\nu}_{emission} = \Delta\tilde{\nu} = \frac{2(\mu_e - \mu_g)^2}{hca^3}\left[\left(\frac{\varepsilon-1}{2\varepsilon+1}\right) - \left(\frac{n^2-1}{2n^2+1}\right)\right] + C \qquad \text{Equation 1}$$

Equation 1 is a classical description of the solvent-dependent shift in the observed Stokes Shift for fluorescent species, modeled as point-dipoles.[3] We had previously shown that the absorption bands of probe **2** in select solvents were not readily affected by solvent polarity, allowing us to simplify Equation 1 to a relationship between the observed fluorescence emission and the solvent polarity (Equation S1).[1]

$$\tilde{\nu}_{\mathrm{em}} = \frac{2(\mu_e - \mu_g)^2}{hca^3}\left[\left(\frac{\varepsilon-1}{2\varepsilon+1}\right) - \left(\frac{n^2-1}{2n^2+1}\right)\right] + C' \qquad \text{Equation S1}$$

Additionally, we also showed that the fluorescence emission of probe **2** was not strongly affected by the refractive index of the solvents. Therefore, we expected that removal of the refractive index term from the permittivity field would not greatly affect the observed emission profile of the fluorophores in a given environment. The resulting truncation of the Lippert-Mataga equation results in equation 2, where we describe the relationship of the observed fluorescence emission wavelength and the local relative permittivity (also referred to as dielectric constant), *ε*.

$$\tilde{\nu}_{\mathrm{em}} = \frac{2(\mu_e - \mu_g)^2}{hca^3}\left(\frac{\varepsilon-1}{2\varepsilon+1}\right) + C_1 \qquad \text{Equation 2}$$

Supplementary Note

We did not observe a good correlation between the absorption bands and the orientation polarizability, suggesting that the environmental sensitivity of these probes is dominated by solvent stabilization of the emissive S_1 excited state (Supplementary Figure 2A, 2B, and Supplementary Table 1). The solvatochromic nature of the fluorophores was weighted more by the excited state dipole moments than the ground state (i.e. solvent reorganization around the molecule was more pronounced for the excited state, so the energy of the ground state destabilization was relatively negligible compared to the energy of the stabilization of the excited state). Additionally, plots of the fluorescence emission bands were weakly correlated to the refractive index term, $f(n)$, of the orientation polarizability (Supplementary Figure 2C). In contrast, the emission was strongly correlated to the dielectric constant term, $f(\varepsilon)$ (See Figure 3C in the main text).

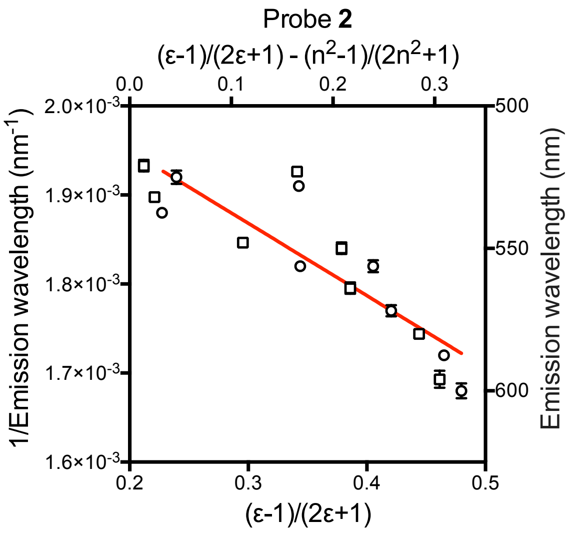

Supplementary Figure 1. The fluorescence emission of AACA probe 2 plotted as a function of the full Lippert-Mataga solvent parameters (☐) or just the dielectric constant parameter alone (○).

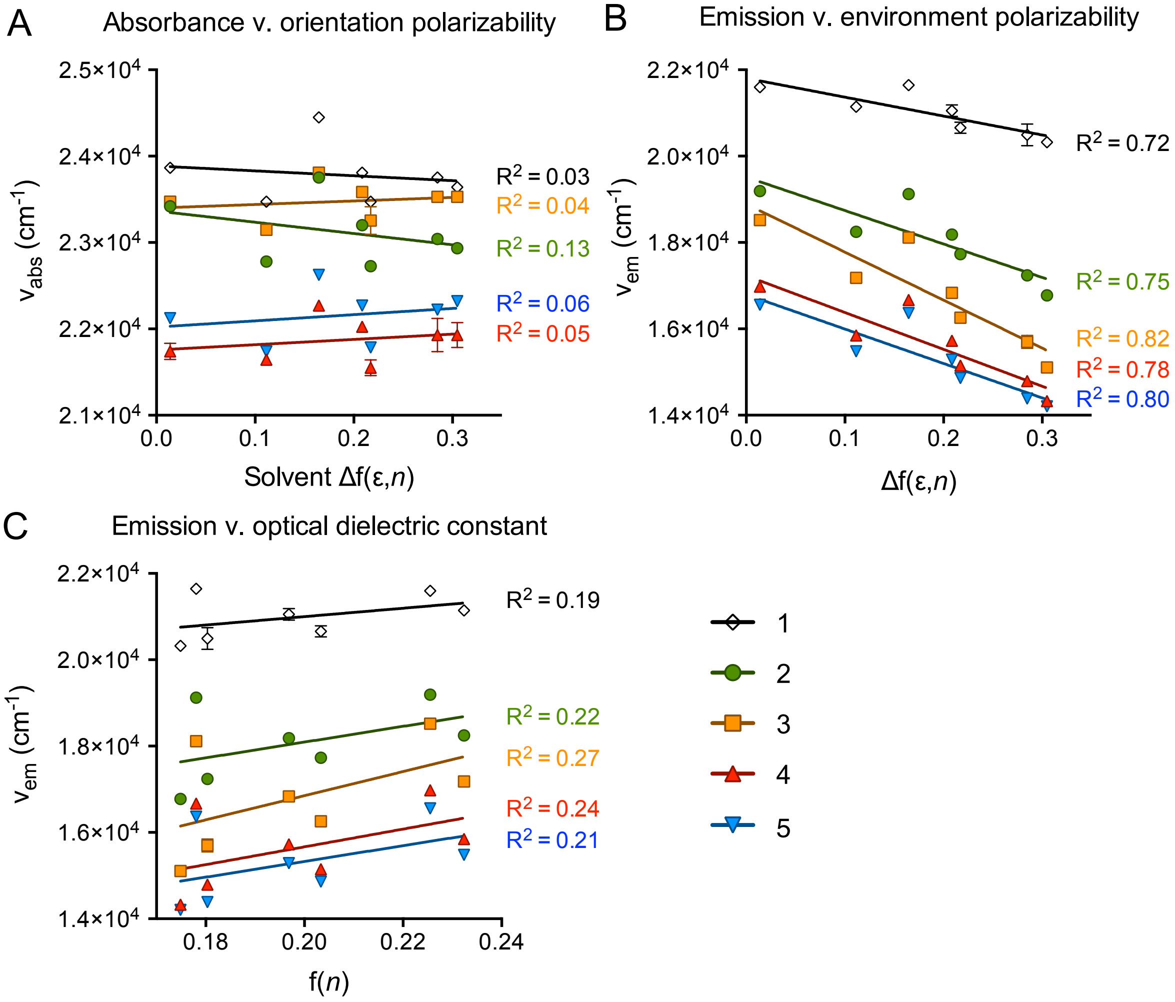

Supplementary Figure 2. Solvatochromic behavior of Probes **1 - 5**. A) Plot showing relationship between observed absorption bands (in wavenumbers) and the solvent permittivity (described by the orientation polarizability, Δf(*ε*,*n*)). B) Plot of the observed fluorescence emission (in wavenumbers) and the solvent orientation polarizability. C) Plot of the observed fluorescence emission (in wavenumbers) and the solvent polarity, f(*n*).

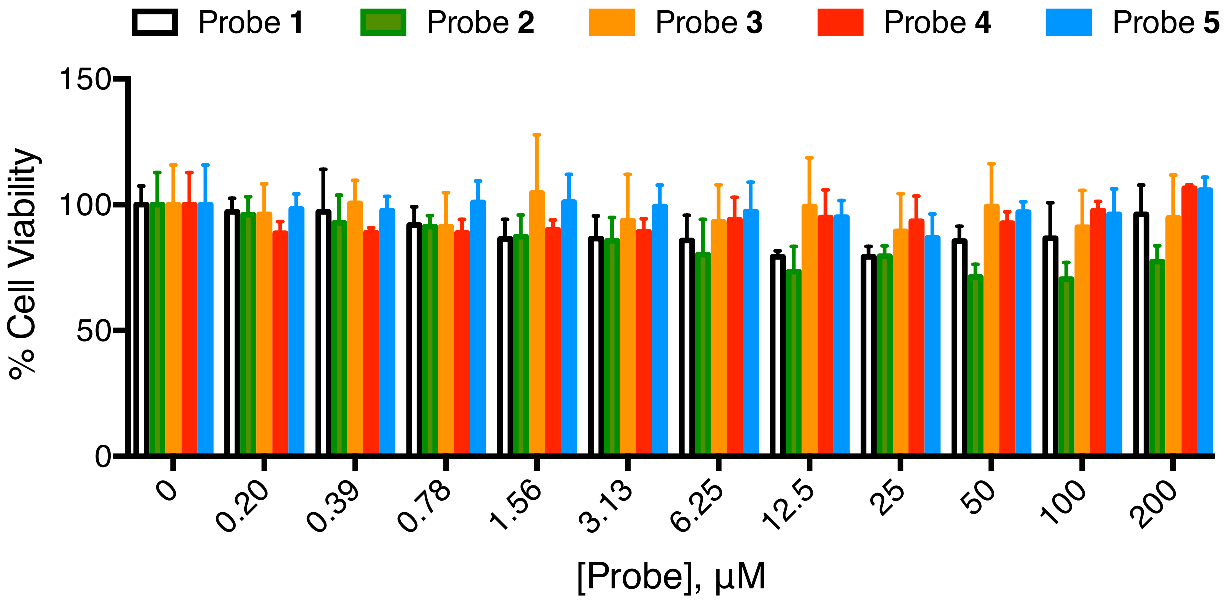

Supplementary Figure 3. Cell viability of SH-SY5Y neuroblastoma cells 24 h after dosing with various concentrations of AACA probes **1 – 5**. Error bars denote standard deviation from the mean, n = 4.

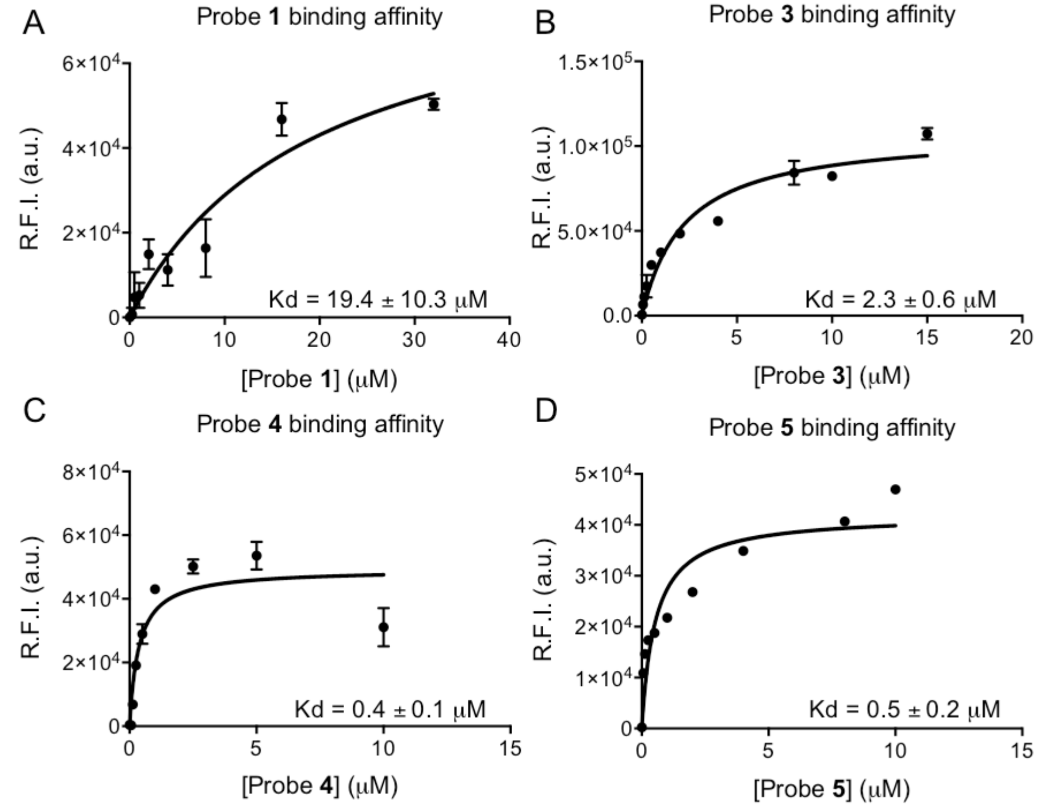

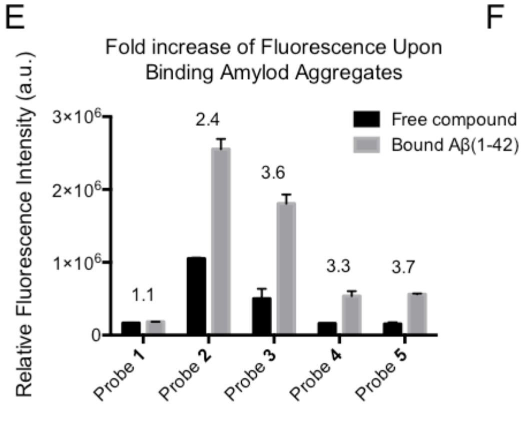

F

AACA	Emission λ_{max} (free/bound)	Relative quantum yield (free/bound)
1	499(4)/(497(5)	0.127(7)/0.132(4)
2	594(2)/543(5)	0.257(4)/0.580(7)
3	610(5)/569(2)	0.178(5)/0.566(7)
4	615(3)/608(3)	0.087(5)/0.185(9)
5	615(3)/612(3)	0.074(6)/0.18(1)

Supplementary Figure 4. Fluorescence emission properties of AACA probes **1**, **3**, **4**, and **5** after binding amyloid aggregates. A – D) Probe binding affinities towards aggregated β-amyloid (1 – 42) (Aβ(1 – 42)) in solution. Binding affinity of AACA probe **2** was previously reported.[1] E) Relative fold increase of fluorescence emission intensity after binding aggregates. F) Tabulated fluorescence emission properties of AACA probes bound to Aβ(1 – 42). Error bars indicate standard deviation for measurements in triplicate.

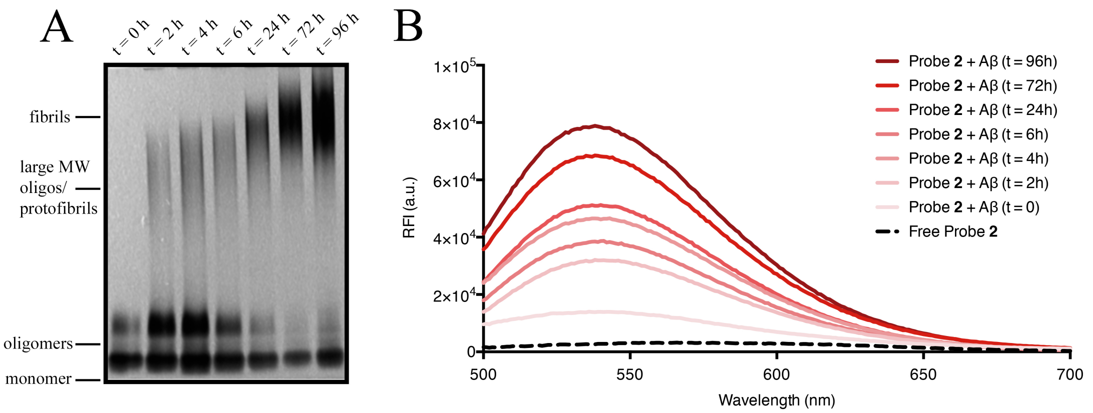

C

Aggregation Time (h)	Avg. λmax Emission (nm)
0	540 ± 4
2	538 ± 1
4	539 ± 4
6	540 ± 2
24	539 ± 2
72	538 ± 4
96	537 ± 3

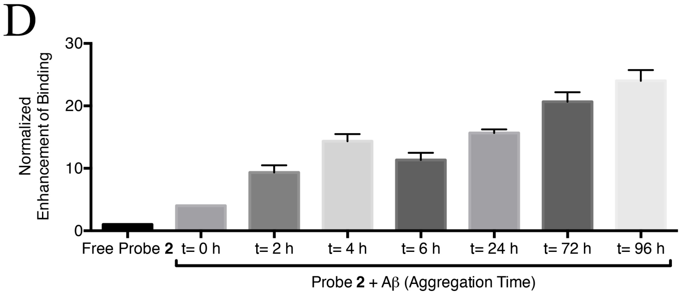

Supplementary Figure 5. Fluorescence emission properties of AACA probe **2** upon incubation with β-amyloid (1–42) peptides at different timepoints of aggregation in solution. A) Silver stained native gel showing the various aggregation species of Aβ(1–42) present versus time incubated at 37°C (0-96 hours). B) Fluorescence emission spectra of AACA probe **2** with or without Aβ(1–42) aggregated species at different timepoints during the aggregation process. C) Tabulated fluorescence emission λ_{max} values of probe **2** with Aβ(1–42) at different aggregation time points. D) Relative fold increase of fluorescence emission λ_{max} intensity upon exposure to Aβ(1–42) aggregates as a function of different aggregation time points. For normalization, the average maximum intensity of the free probe (i.e., in the absence of Aβ(1–42)) was set to 1. Data is presented as mean values ± standard deviation for measurements preformed in at least triplicate.

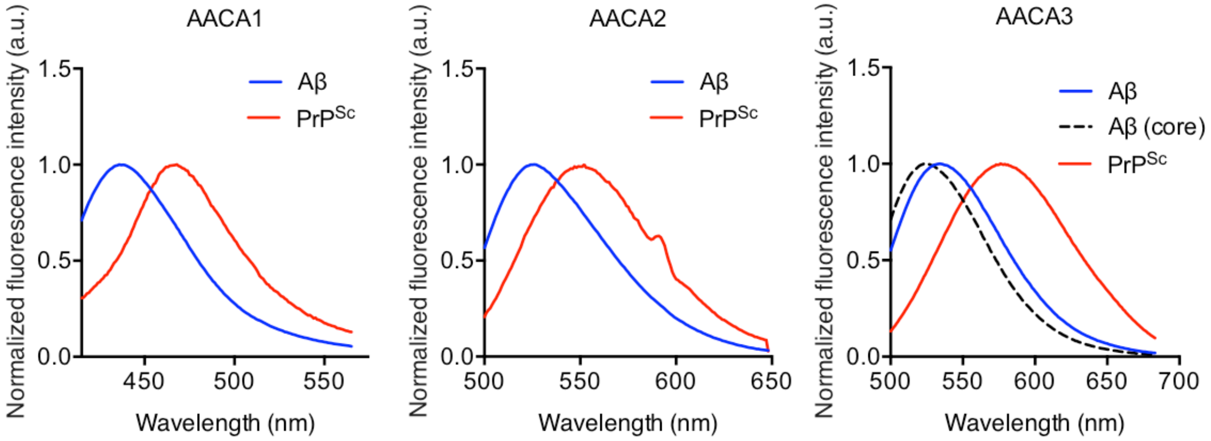

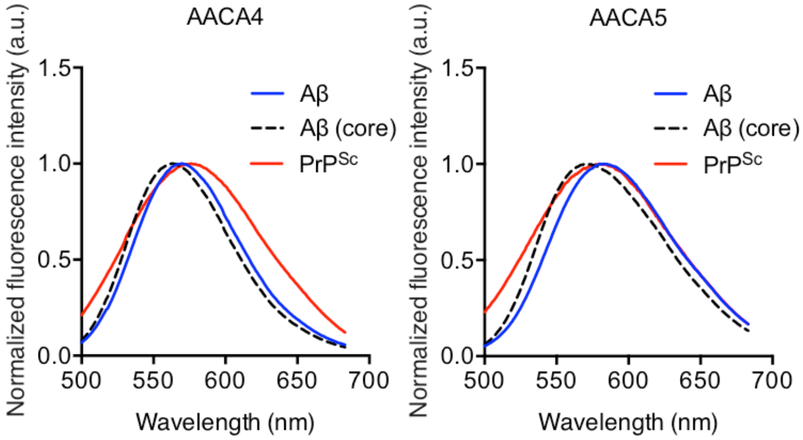

AACA	Aβ	Aβ (core)	PrP^{Sc}
1	437(2)	N/A	467(2)
2	525(2)	N/A	550(3)
3	536(9)	524(2)	577(3)
4	570(6)	564(3)	576(3)
5	583(4)	571(5)	580(4)

Supplementary Figure 6. Fluorescence emission spectra of β-amyloid and prion plaques in neurodegenerative tissue from Tg mice stained with AACA probes **1** – **5**. The β-amyloid plaques stained with probes **3** – **5** were examined using an ROI tool in FluoView to separate the core (black dashed lines) from the rest of the plaque. Emission λ_{max} values with recorded error were tabulated in nm.

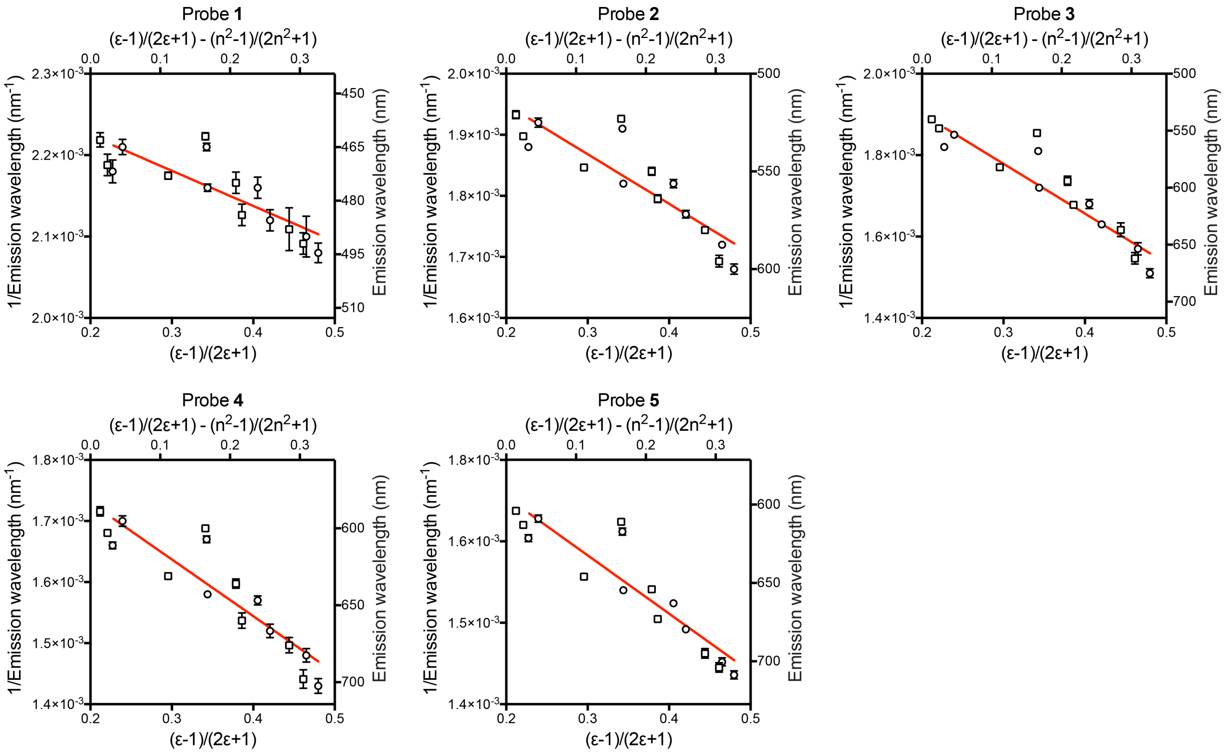

Supplementary Figure 7. Calibration curves for spectroscopic ruler measurements. Solvatochromic behavior of AACA probes **1** – **5** described by Equation 2 was used to extrapolate binding pocket permittivities of the probes on various amyloid aggregates.

Supplementary Table 1. Fluorescence absorption and emission properties of AACA **1** – **5** in aprotic solvents.

	Fluorescence properties ($\lambda_{max, abs}$ (nm) / $\lambda_{max, em}$ (nm), ε (x10^3 M^{-1} cm^{-1}))				
Solvent	AACA **1**	ANCA **2**	AACA **3**	AACA **4**	AACA **5**
Toluene	419/463, 78.6	427/521, 48.6	426/540, 62.1	460/589, 51.1	452/604, 65.8
Diethyl ether	409/462, 58.9	421/523, 49.4	420/552, 51.1	449/600, 51.2	442/611, 69.1
Anisole	426/473, 46.8	439/548, 47.4	432/582, 49.7	462/631, 47.7	460/646, 55.7
THF	420/475, 51.9	431/550, 55.5	424/594, 54.8	454/636, 47.7	449/654, 50.3
DCM	426/484, 81.0	440/564, 67.2	430/615, 64.6	464/660, 40.1	459/673, 67.3
Acetone	421/488, 45.6	434/580, 44.5	425/637, 56.9	456/676, 43.9	450/695, 57.7
Acetonitrile	423/492, 86.6	436/596, 55.1	425/662, 69.9	456/798, 63.2	448/704, 63.2

Supplementary Table 2. Correlation values of solvatochromic behavior. Slope values in units of (cm^{-1}), R^2 values are listed next to each slope.

Solvatochromicsm correlation values (slops of Lippert-Mataga plots)

Probe	Stokes v. Δf(ε, n)	R^2	Absorbance v. Δf(ε, n)	R^2	Emission v. Δf(ε, n)	R^2	Emission v. Δf(n)	R^2	Emission v. Δf(ε)	R^2
1	3814 ± 557	0.90	-565 ± 1454	0.03	-4379 ± 1221	0.72	9727 ± 9073	0.19	-5649 ± 1182	0.82
2	6443 ± 1122	0.89	-1306 ± 1519	0.13	-7749 ± 1999	0.75	18264 ± 15411	0.22	-9917 ± 1927	0.84
3	11535 ± 1627	0.90	408 ± 949	0.04	-11128 ± 2329	0.82	27965 ± 20430	0.27	-14107 ± 2074	0.90
4	9152 ± 1101	0.93	612 ± 1055	0.06	-8540 ± 1923	0.80	20585 ± 18255	0.24	-10893 ± 1725	0.89
5	8699 ± 1032	0.93	720 ± 1336	0.06	-797 ± 1891	0.78	18225 ± 15607	0.21	-10256 ± 1671	0.88

Supplementary Table 3. Change in dipole moment as a function of the solvent environment, from DFT calculations.

		Calculated $(\mu_e - \mu_g)^2$ (Debyes^2)				
Solvent	Dielectric Constant (ε)	Probe **1**	Probe **2**	Probe **3**	Probe **4**	Probe **5**
Water	80.400	49.758910	200.05270	230.20480	255.86240	286.70960
Diemethyl Sulfoxide (DMSO	46.826	48.177480	194.49090	220.25830	245.75580	275.28780
Acetonitrile (ACN)	36.640	46.977320	190.24680	215.32040	239.07340	268.77960
Acetone	21.010	43.375400	178.00900	198.05780	217.21160	246.70990
Anisole	4.300	22.099400	100.60090	89.44431	98.79167	113.77850
Toluene	2.379	10.824100	59.79929	41.33461	41.02915	51.95671
1,4-Dioxane	2.250	9.541921	54.89328	35.82023	35.82501	45.22159

Supplementary Table 4. Torsion angle (ϕ) defined as the dihedral angle between the X-C-C-C atoms (X = N, O or S) for Probes **3** – **5** in the ground state (GS) and excited state (ES) fully optimized structures at DFT level (all values in degrees), as a function of the solvent.

	ϕ (GS) / °	ϕ (ES) / °		ϕ (GS) / °	ϕ (ES) / °
**Vacuum**			**Anisole**		
NMe	-48.68	-24.87	NMe	-45.72	-22.46
O	-3.22	-0.03	O	-1.84	0.61
S	-27.90	-1.19	S	-24.81	-0.62
**DMSO**			**Acetonitrile**		
NMe	-43.84	-20.79	NMe	-43.99	-20.68
O	-0.92	-0.08	O	-2.00	0.17
S	-23.28	-0.43	S	-23.40	0.27
**Toluene**			**Acetone**		
NMe	-46.73	-23.65	NMe	-44.30	-20.98
O	-2.31	0.74	O	-3.25	0.12
S	-26.24	-0.08	S	-23.99	0.26

Supporting references

1. Cao, K. *et al.* Aminonaphthalene 2-cyanoacrylate (ANCA) probes fluorescently discriminate between amylid-β and prion plaques in brain. *J. Am. Chem. Soc.* **134,** 17338–17341 (2012).
2. Chang, W. M. *et al.* ANCA: A Family of Fluorescent Probes that Bind and Staing Amyloid Plaques in Human Tissue. *ACS Chem. Neurosci.* **2,** 249–255 (2011).
3. Lakowicz, J. R. *Principles of fluorescence spectroscopy*. *Principles of Fluorescence Spectroscopy* (2006).

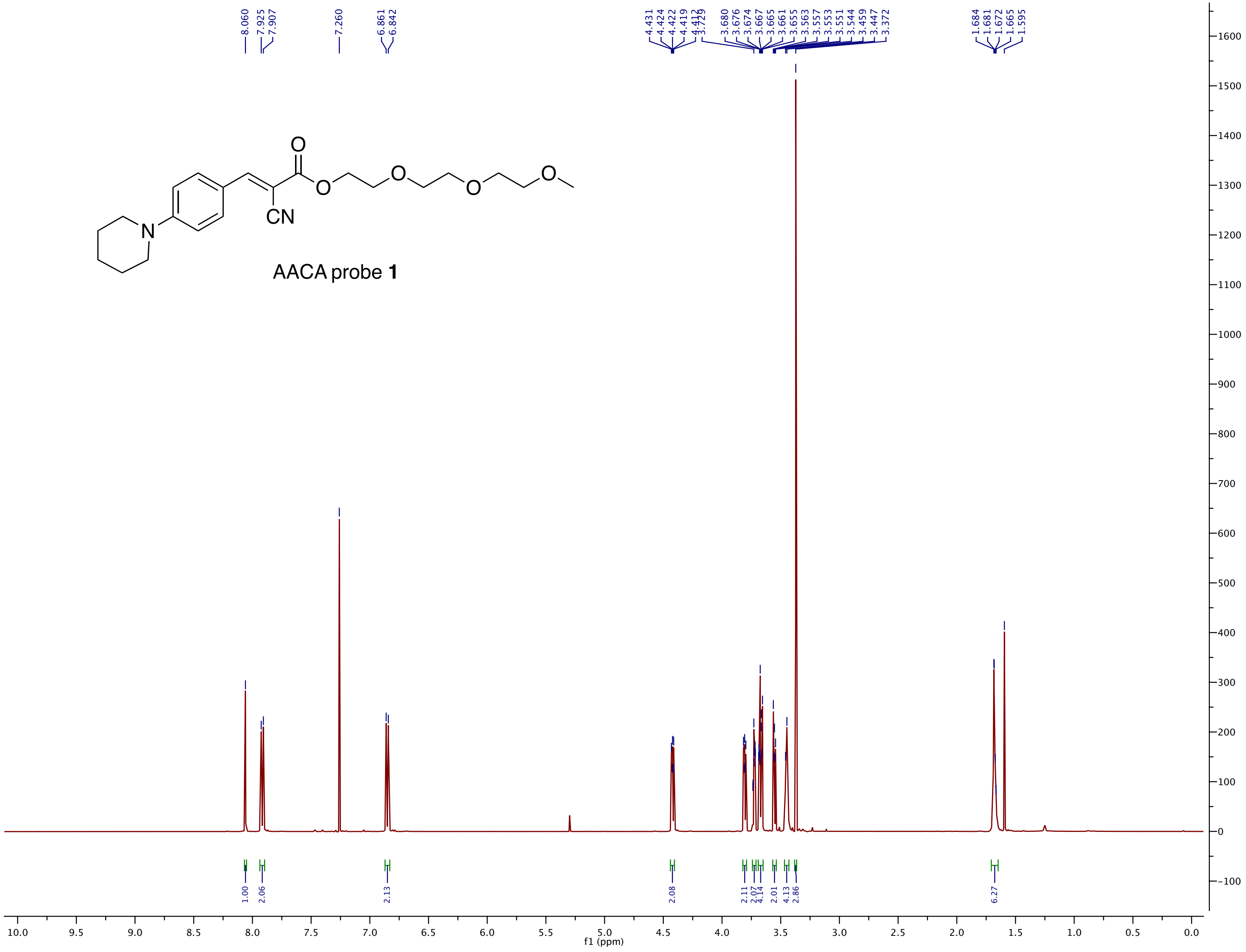
O
O
O
O
O
N
CN
8.060
7.925
7.907
7.260
6.861
6.842
4.431
4.424
4.422
4.419
3.729
3.680
3.676
3.674
3.667
3.665
3.661
3.655
3.563
3.557
3.553
3.551
3.544
3.459
3.447
3.372
1.684
1.681
1.672
1.665
1.595
1.00
2.06
2.13
2.08
2.11
2.07
4.14
2.01
4.13
2.86
6.27
f1 (ppm)

AACA probe **1**

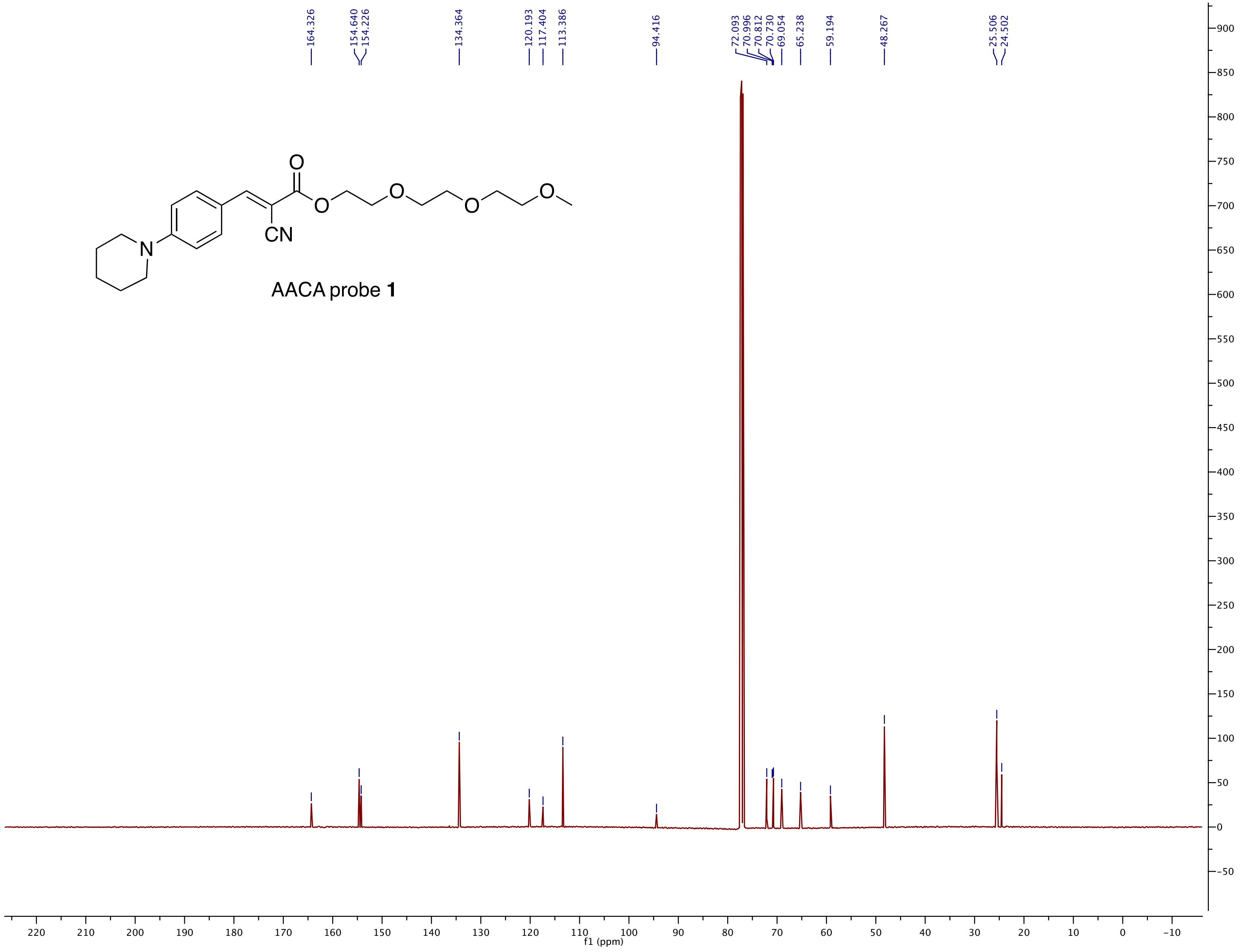
164.326
154.640
154.226
134.364
120.193
117.404
113.386
94.416
72.093
70.996
70.812
70.730
69.054
65.238
59.194
48.267
25.506
24.502
AACA probe 1
f1 (ppm)

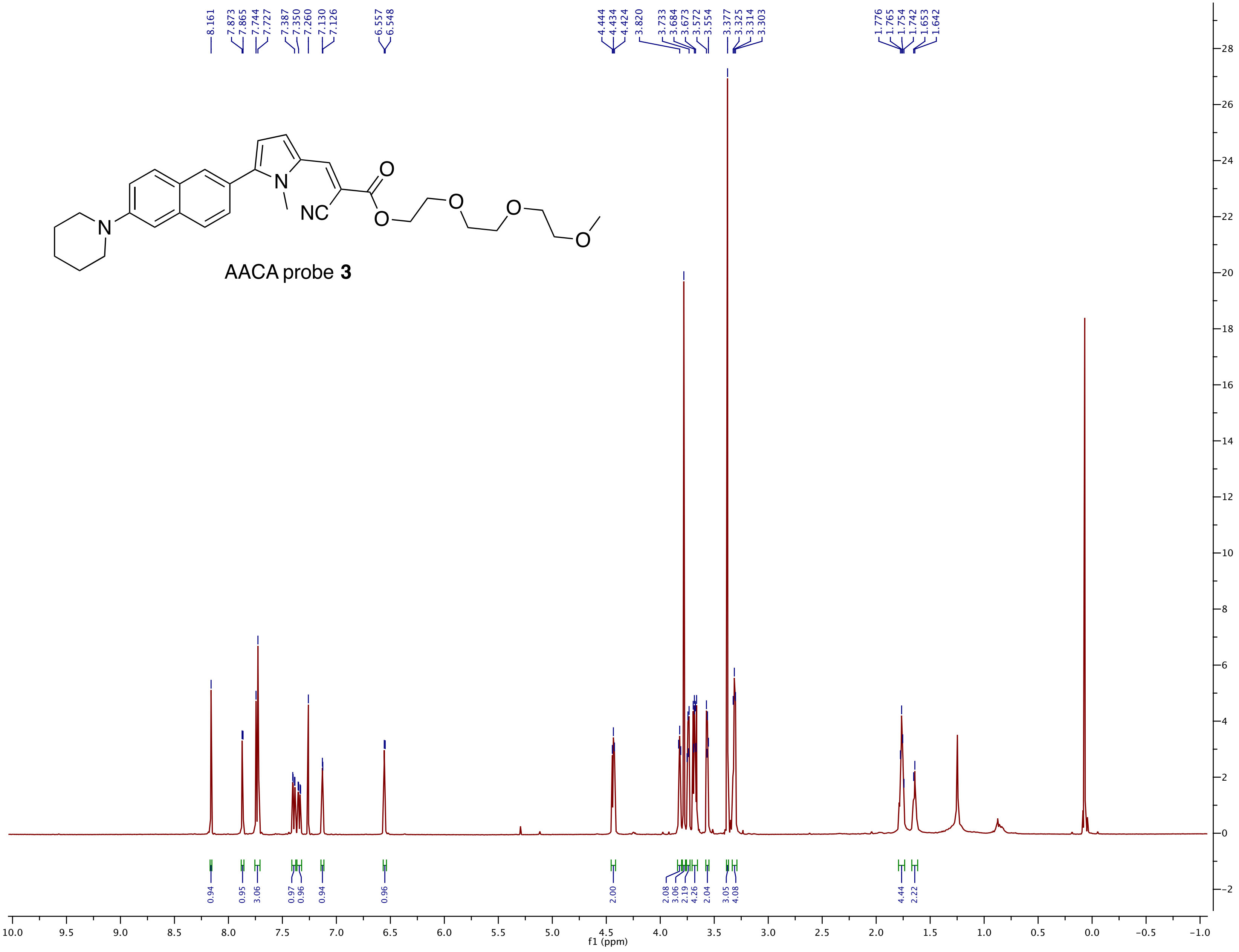
AACA probe **3**
f1 (ppm)

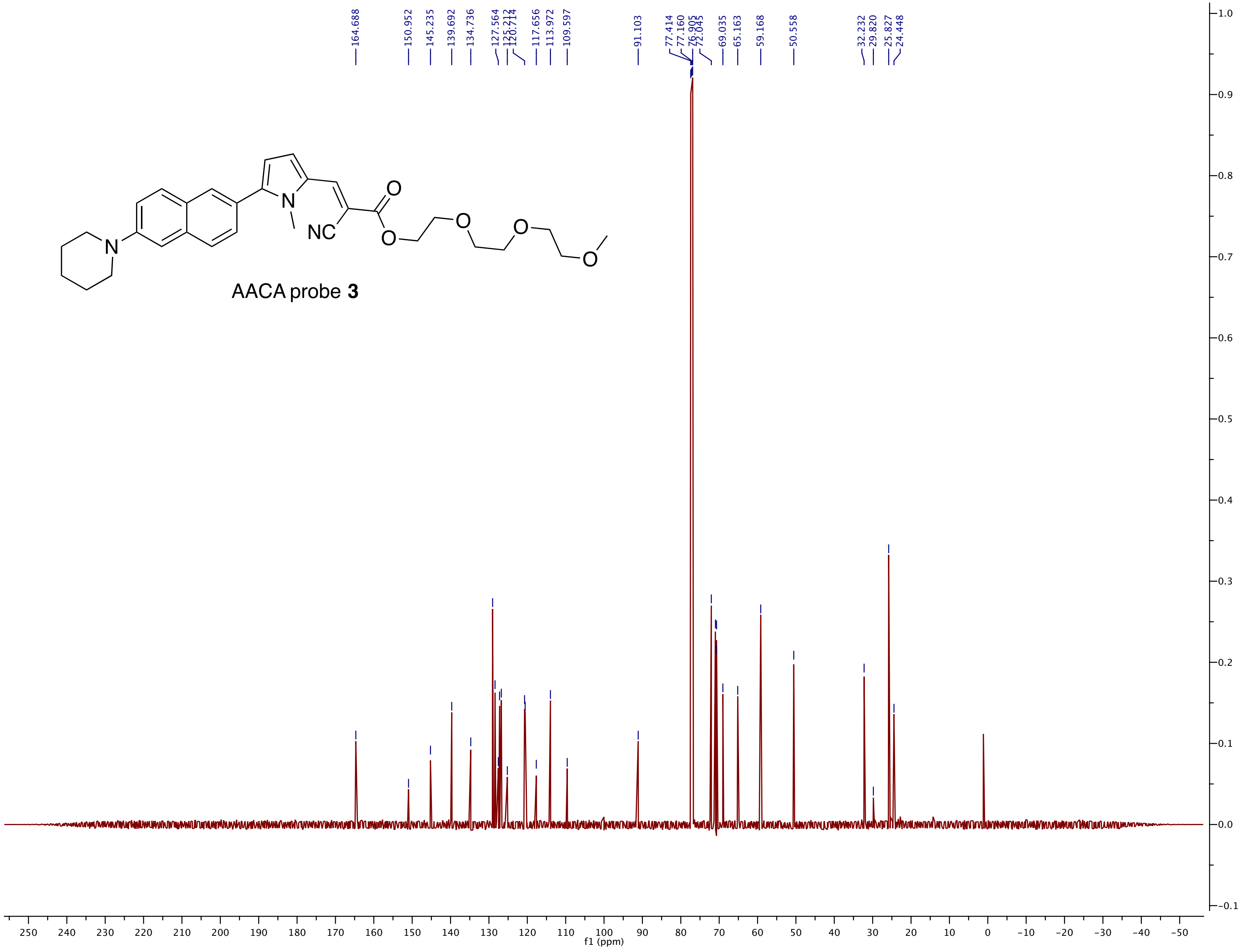

164.688
150.952
145.235
139.692
134.736
127.564
125.212
120.714
117.656
113.972
109.597
91.103
77.414
77.160
76.905
72.045
69.035
65.163
59.168
50.558
32.232
29.820
25.827
24.448
NC
AACA probe **3**
f1 (ppm)

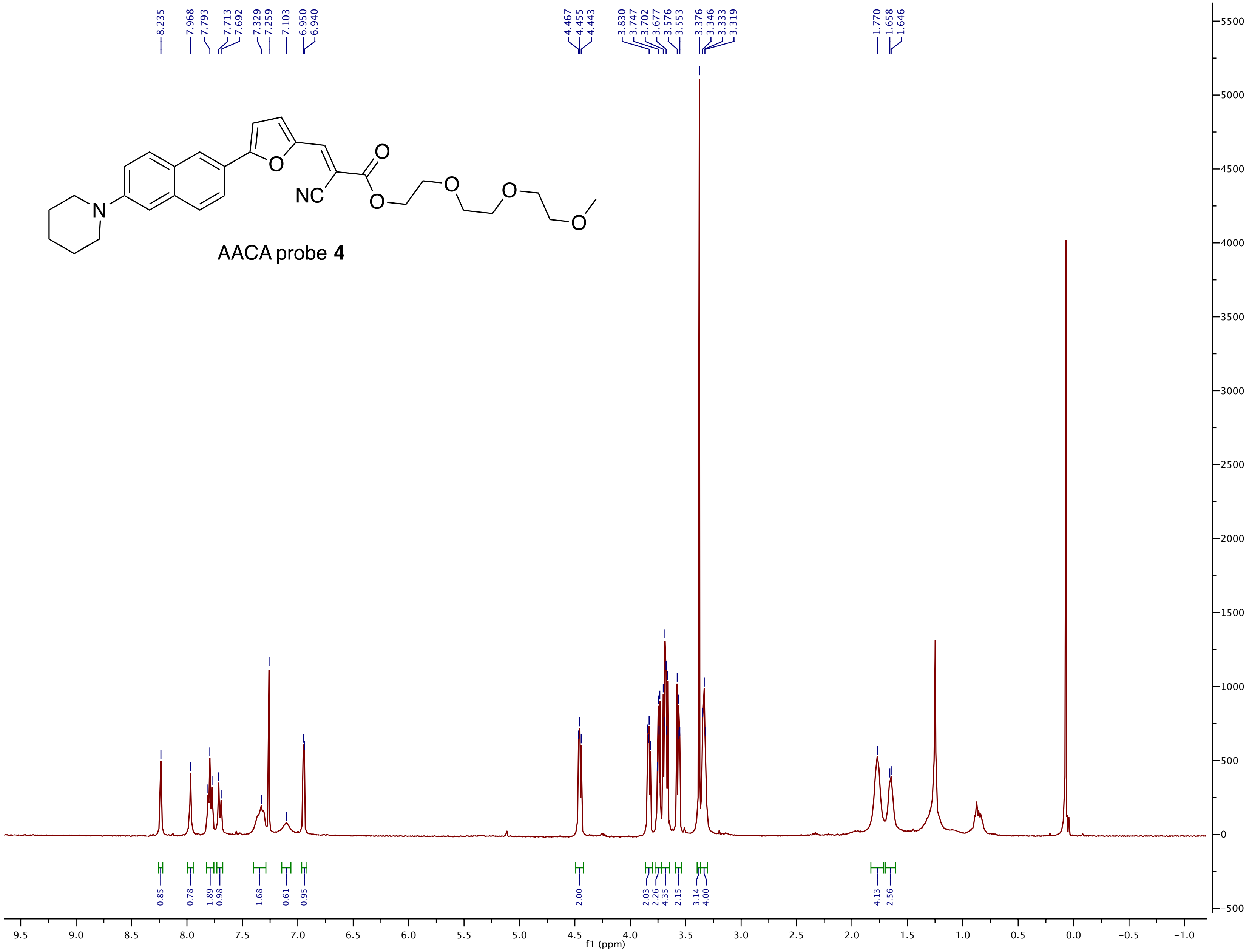

8.235
7.968
7.793
7.713
7.692
7.329
7.259
7.103
6.950
6.940
4.467
4.455
4.443
3.830
3.747
3.702
3.677
3.576
3.553
3.376
3.346
3.333
3.319
1.770
1.658
1.646
O
O
NC
O
O
O
O
N
AACA probe 4
0.85
0.78
1.89
0.98
1.68
0.61
0.95
2.00
2.03
2.26
4.35
2.15
3.14
4.00
4.13
2.56
f1 (ppm)

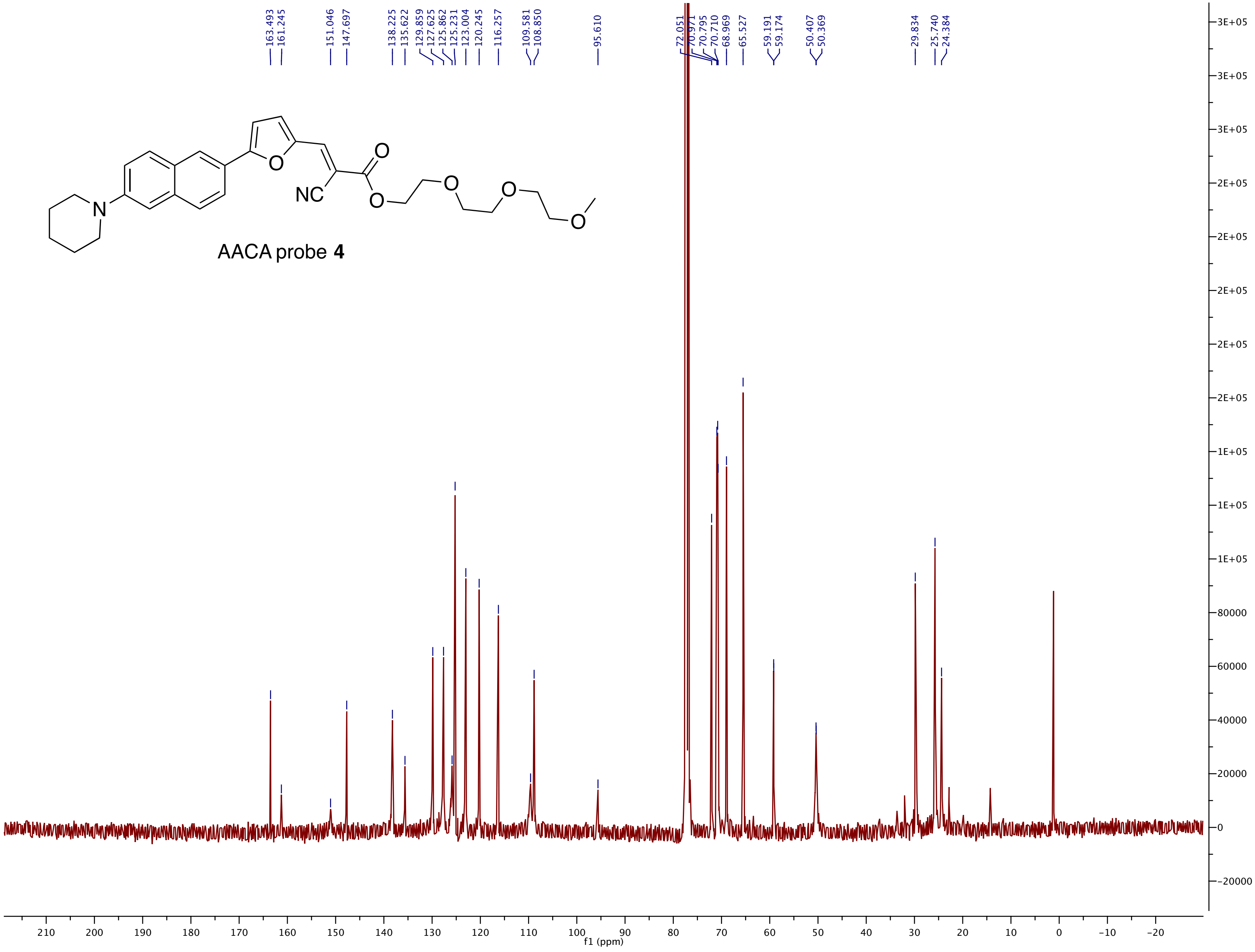
163.493
161.245
151.046
147.697
138.225
135.622
129.859
127.625
125.862
125.231
123.004
120.245
116.257
109.581
108.850
95.610
72.051
70.971
70.795
70.710
68.969
65.527
59.191
59.174
50.407
50.369
29.834
25.740
24.384
NC
AACA probe 4
f1 (ppm)

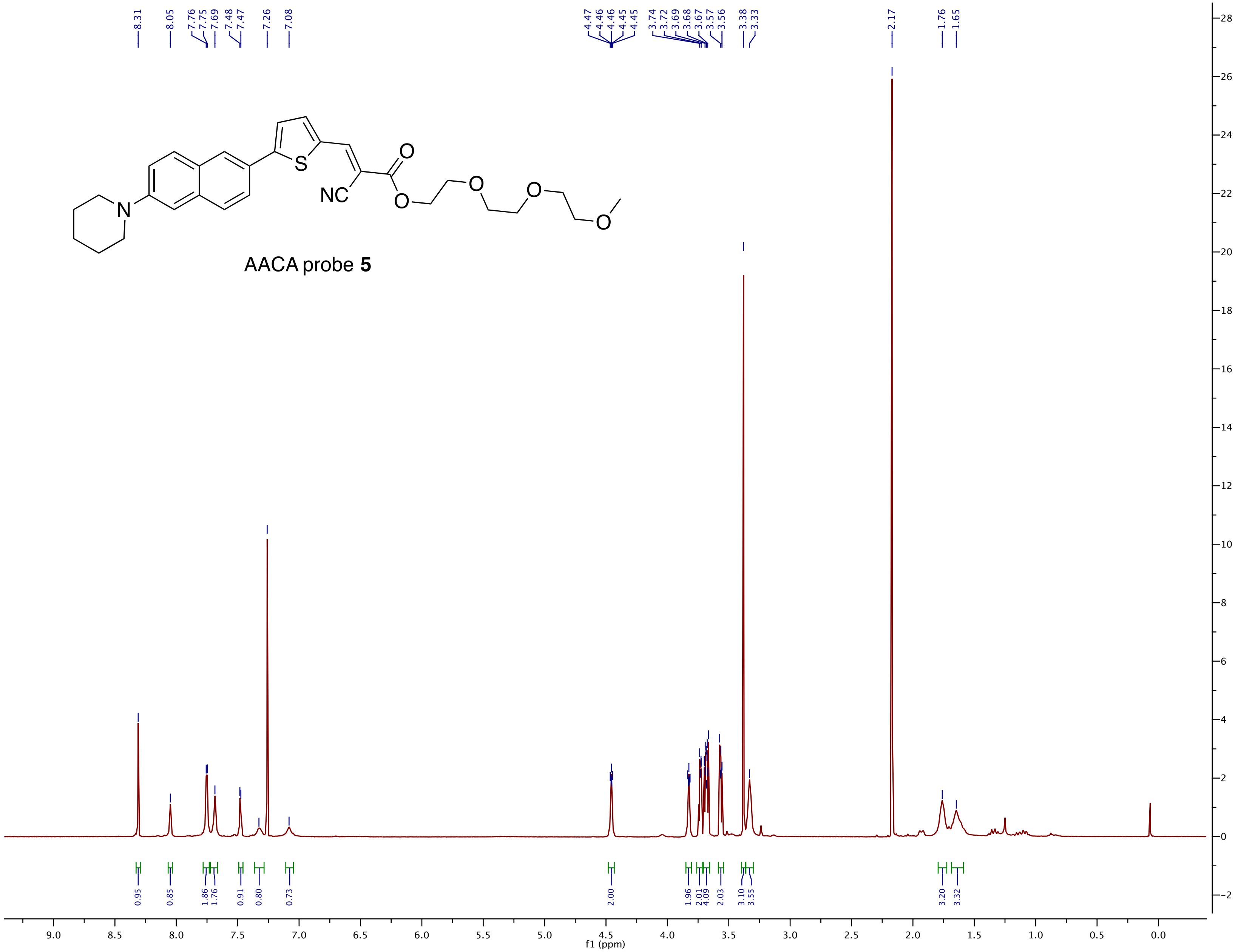
8.31
8.05
7.76
7.75
7.69
7.48
7.47
7.26
7.08
4.47
4.46
4.46
4.45
4.45
3.74
3.72
3.69
3.68
3.67
3.57
3.56
3.38
3.33
2.17
1.76
1.65
NC
S
O
N
AACA probe 5
0.95
0.85
1.86
1.76
0.91
0.80
0.73
2.00
1.96
2.01
4.09
2.03
3.10
3.55
3.20
3.32
f1 (ppm)

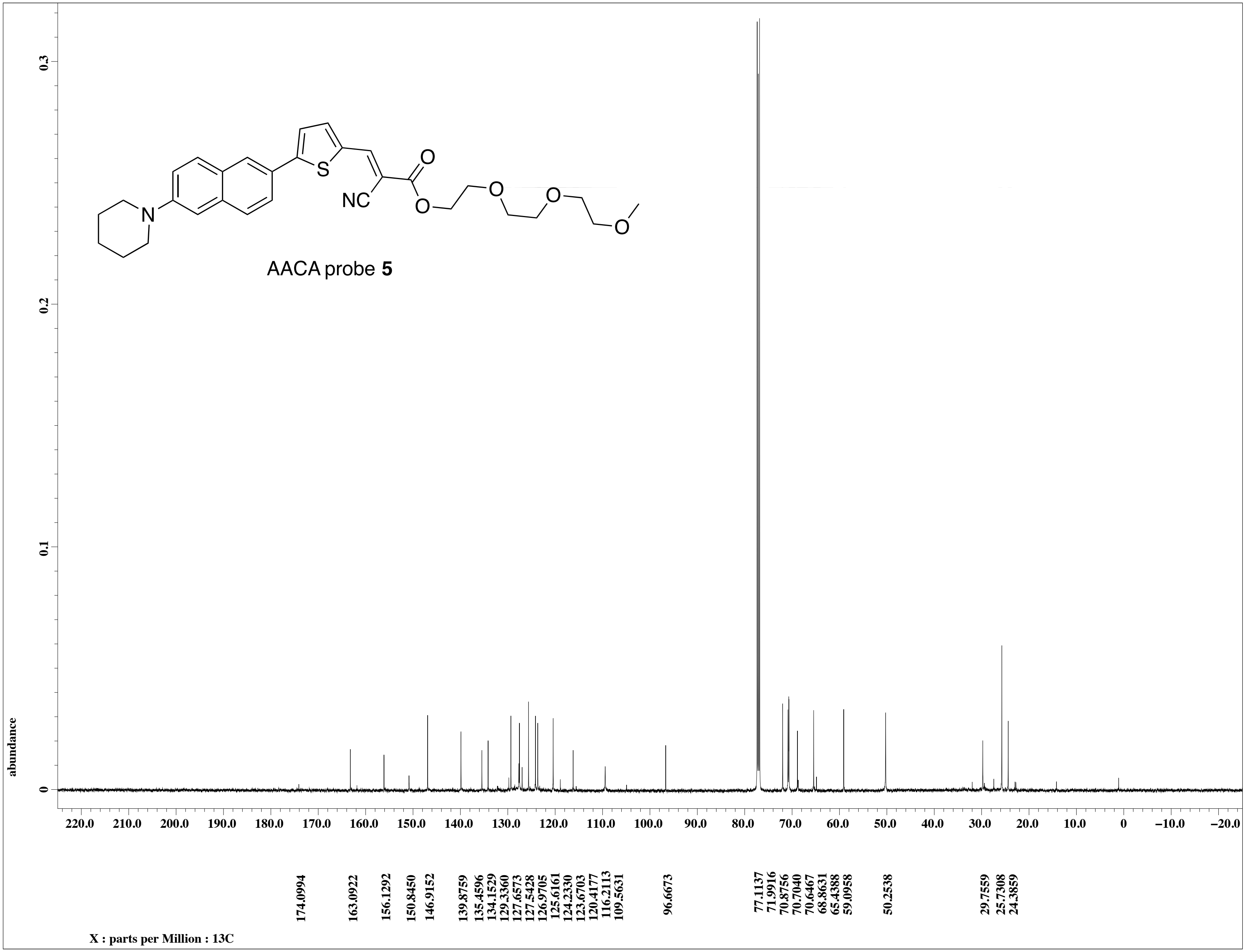

NC
S
O
O
O
O
O
N
AACA probe **5**
abundance
0.3
0.2
0.1
0
220.0 210.0 200.0 190.0 180.0 170.0 160.0 150.0 140.0 130.0 120.0 110.0 100.0 90.0 80.0 70.0 60.0 50.0 40.0 30.0 20.0 10.0 0 −10.0 −20.0
174.0994
163.0922
156.1292
150.8450
146.9152
139.8759
135.4596
134.1529
129.3360
127.6573
127.5428
126.9705
125.6161
124.2330
123.6703
120.4177
116.2113
109.5631
96.6673
77.1137
71.9916
70.8756
70.7040
70.6467
68.8631
65.4388
59.0958
50.2538
29.7559
25.7308
24.3859
X : parts per Million : 13C
